# KCMF1-mediated influenza A virus PB1 ubiquitination at K653 regulates viral replication

**DOI:** 10.1128/jvi.00481-26

**Published:** 2026-06-15

**Authors:** Xianfeng Hui, Xiaowei Tian, Chang Xue, Shihuan Ding, Jiyan Cui, Aiping Sun, Wei Lu, Yunwei Lou, Shaoju Qian, Tiesuo Zhao, Liangwei Duan, Hui Wang

**Affiliations:** 1 Department of Immunology, School of Basic Medical Sciences, Henan Medical University 91593, Xinxiang, China; 2 Henan Key Laboratory of Immunology and Targeted Drug, Henan Medical University 91593, Xinxiang, China; 3 Xinxiang Engineering Technology Research Center of Immune Checkpoint Drug for Liver-Intestinal Tumors, Henan Medical University 91593, Xinxiang, China; 4 Department of Pathogenic Biology, School of Basic Medical Sciences, Henan Medical University 91593, Xinxiang, China; 5 Henan Collaborative Innovation Center of Molecular Diagnosis and Laboratory Medicine, School of Medical Technology, Henan Medical University 91593, Xinxiang, China; Fred Hutchinson Cancer Center Vaccine and Infectious Disease Division, Seattle, Washington, USA

**Keywords:** influenza A virus, PB1 protein, E3 ubiquitin ligase, ubiquitin, K653 site, immune defense

## Abstract

Ubiquitination is a pivotal regulatory mechanism in host-virus interactions, playing essential roles in both antiviral defense and viral immune evasion, with E3 ubiquitin ligases serving as critical regulatory nodes. In this study, we systematically evaluated 26 candidate host E3 ubiquitin ligases for their involvement in influenza A virus (IAV) infection. Among these, potassium channel modulatory factor 1 (KCMF1) was identified as a novel negative regulator of IAV replication *in vitro* and *in vivo*. Mechanistically, KCMF1 interacts with the viral polymerase subunit PB1 and mediates its ubiquitination at lysine 653 (K653), triggering proteasomal degradation and consequent impairment of polymerase activity. A recombinant PR8 (H1N1) virus carrying a K653R substitution in PB1 exhibits enhanced replication and increased pathogenicity in both cells and mice. Furthermore, the inhibitory effect of KCMF1 on PR8 virus replication is dependent on the K653 residue of PB1. Importantly, viruses harboring the PB1 K653 mutation display marked resistance to favipiravir (T-705), an inhibitor of viral RNA-dependent RNA polymerase, suggesting that mutations at this site may influence antiviral drug sensitivity in circulating strains, and have potential implications for clinical treatment and viral surveillance. In conclusion, our findings identify KCMF1 as a host restriction factor that suppresses IAV replication via ubiquitination of PB1 at K653.

**IMPORTANCE:**

Influenza A virus (IAV) continues to pose a major global health threat, and host factors that regulate viral replication are critical for understanding pathogenesis and guiding antiviral interventions. Here, we identify KCMF1 as a host E3 ubiquitin ligase that restricts IAV replication by promoting ubiquitination-dependent degradation of the viral polymerase subunit PB1. We further define lysine 653 (K653) of PB1 as a critical residue for this regulatory mechanism. Notably, mutation at this site enhances viral replication and pathogenicity while conferring resistance to favipiravir, a clinically approved inhibitor of viral RNA-dependent RNA polymerase. Collectively, these findings provide new mechanistic insights into host–virus interactions and highlight important considerations for antiviral drug use and surveillance of emerging viral variants.

## INTRODUCTION

Influenza A virus (IAV) is responsible for an estimated 290,000–650,000 deaths globally each year (1) and was identified by the World Health Organization (WHO) as one of the top 10 global health threats at the start of 2019. Despite significant advancements in influenza virus infection through vaccines and antiviral drugs, the effectiveness of these interventions is compromised by the virus’s high mutation rate and the emergence of drug-resistant strains (2–4). The influenza virus relies not only on its own proteins but also on host cell proteins and their associated mechanisms to complete its life cycle (5). Consequently, gaining a deeper understanding of how viral components interact with and exploit host molecules is crucial for elucidating the mechanisms of pathogenesis and identifying potential targets for antiviral therapy.

Protein ubiquitination is an important interactive post-translational modification (6). Since the seminal work of Shaw et al., which highlighted ubiquitin as a critical host-encoded protein in influenza virus particles (7), studies on the ubiquitination of IAV proteins have increasingly gained prominence. E3 ubiquitin ligases serve a dual function in the context of viral-host interactions. On the one hand, they are employed by viruses to specifically degrade host antiviral factors, thereby facilitating immune evasion (8). On the other hand, the host can also utilize the ubiquitin-proteasome system to degrade some viral proteins, which diminishes viral infectivity (9). Collectively, these processes underscore the significant role of ubiquitination in both host immunity and viral pathogenicity, illustrating its dual nature as a double-edged sword for both the virus and the host.

The viral ribonucleoprotein (vRNP) complex is integral to the life cycle of IAV, as it catalyzes the transcription and replication of the viral genome within the nucleus of infected cells (10). This complex comprises individual viral RNA segments, nucleoprotein (NP), and three polymerase subunits: polymerase basic protein 1 (PB1), polymerase basic protein 2 (PB2), and polymerase acidic protein (PA) (11). As the smallest functional unit of viral genome transcription and replication, the activity of vRNP is dependent on host factors, making it a key target in studies of virus–host interactions and antiviral drug development (12).

In this study, we identify potassium channel modulatory factor 1 (KCMF1) as an E3 ubiquitin ligase that negatively regulates IAV replication and further investigate its interaction with the viral polymerase subunit PB1 and the underlying molecular mechanisms.

## MATERIALS AND METHODS

### Cells

Adenocarcinoma human alveolar basal epithelial cells (A549) were purchased from ATCC and maintained in F12 media (HyClone, Beijing, China) supplemented with 10% fetal bovine serum (FBS). Human embryonic kidney (HEK293T) cells, a human cervical cancer cell line (HeLa), and Madin–Darby canine kidney (MDCK) cells were purchased from American Type Culture Collection (ATCC; Manassas, VA, USA) and maintained in Dulbecco’s modified Eagle’s medium (DMEM; HyClone, Beijing, China) supplemented with 10% FBS (Biological Industries, BioInd). The cells were cultured at 37°C in a 5% CO_2_ humidified atmosphere.

### Viruses and reverse genetics

The influenza virus used in this study was A/Puerto Rico/8/34 (H1N1) (PR8) strain. The influenza virus was grown in 9-day-old embryonated chicken eggs. Recombinant viruses were created using the genetic framework of the wild-type (WT) A/Puerto Rico/8/34 (H1N1; PR8) virus through an eight-plasmid reverse genetics system, as described in earlier studies (1). Eight segments of A/Puerto Rico/8/34 (H1N1; PR8) were cloned and introduced into the pHW2000 vector by our research team. The mutant PB1 gene at base position 1958 was produced using PCR-based site-directed mutagenesis techniques. To verify the absence of unwanted mutations, all constructs underwent sequencing. Recombinant viruses were propagated through single passages in embryonic chicken eggs. To ensure there were no additional mutations, virus stocks of both WT and recombinant viruses were also sequenced.

### Antibodies and reagents

Rabbit anti-influenza A virus nucleoprotein monoclonal antibody (Ab; GTX636247) and rabbit anti-influenza A virus M1 (matrix protein) polyclonal Ab (GTX125928) were purchased from GeneTex (Irvine, CA, USA). Mouse anti-flag tag (cat. no. AE005) and anti-HA tag (cat. no. AE008) monoclonal Abs were purchased from ABclonal Technology (Wuhan, China). A rabbit anti-KCMF1 (YT7292) polyclonal Ab was purchased from ImmunoWay Biotechnology Company (TX, USA). The molecular weight was evaluated with a pre-stained protein ladder, 10-180 kDa (26616, ThermoFisher, IL, USA). Horseradish peroxidase-conjugated anti-mouse (AS003) and anti-rabbit (AS014) secondary Abs were purchased from ABclonal Technology. The CoraLite 594-conjugated goat anti-mouse (SA00013-3) secondary Ab was purchased from Proteintech.

Chloroquine (CQ; cat. no. HY-17589A) and MG132 (cat. no. HY-13259) were purchased from MedChemExpress. Trypsin and N-tosyl-L-phenylalanine chloromethyl ketone (TPCK) treated (cat. no. WBC-LS003740) were purchased from Worthington.

### Transfections

Plasmid and siRNA transfection of A549 cells was performed via the TransIT-X2 Dynamic Delivery System (Mirus, Madison, USA) according to the manufacturer’s instructions. Plasmid transfection into HEK293T cells was carried out using PEI solution (Wuhan, China).

### RNA isolation and quantitative real-time PCR

Total RNA isolation and quantitative real-time PCR (qRT-PCR) were carried out via procedures described previously (13). Briefly, RNA was extracted via TRIzol (Thermo Fisher Scientific, Waltham, MA, USA) according to the manufacturer’s protocol. Immediately after that, RNA was transcribed via reverse transcriptase (AMV XL; TaKaRa Bio, Tokyo, Japan). qRT-PCR was performed on an ABI 7500 Fast Real-Time PCR System (Applied Biosystems, Foster City, CA). The mRNA levels of various genes were calculated after normalization to actin level. The primer sequences used for qRT-PCR were listed in Table S3.

### Cytotoxicity assays

The assessment of A549 cell viability after siRNA transfection was conducted using a CCK-8 assay (1), following the instructions provided by the manufacturer (GlpBio, Shanghai, China). Initially, the cells were plated in a 96-well plate and were transfected with siRNA upon reaching around 80% confluency. After 24 h of post-transfection, the CCK-8 reagent was introduced, and the absorbance was measured at 450 nm after a 2 h incubation period at 37°C.

### 50% tissue culture infectious dose (TCID_50_) assays

TCID_50_ assays were performed as previously described (1). Confluent MDCK cells seeded in 96-well plates were infected with the properly diluted virus in DMEM and incubated at 37°C for 1 h. To eliminate unbound virus, the cells were rinsed twice with phosphate-buffered saline (PBS) from HyClone. Following this, the cells received a layer of DMEM supplemented with 1% bovine serum albumin and 3 μg/mL of TPCK-treated trypsin. After 48 h of incubation at 37°C, hemagglutination was assessed by mixing 50 μL of the tissue culture supernatant with 1% turkey erythrocytes. The TCID_50_ value was determined using the Karber method. For processing the lung tissue of mice, whole lung specimens from each mouse were homogenized in 2 mL of PBS, and the supernatant was collected after spinning down at 4°C.

### Polymerase activity assay

A dual-luciferase reporter assay system (Promega, Madison, Wisconsin, USA) was used to compare the polymerase activities of different viral RNP complexes as reported previously (13). HEK293T cells were transfected with pcDNA3.1 constructs expressing viral PB2, PB1 (wild-type or mutant), PA, NP, and the pLuci luciferase reporter plasmid (0.3 μg each), together with the internal control plasmid pRL-TK (0.01 μg), using Lipofectamine 2000 (Invitrogen, Carlsbad, CA, USA). To ensure equal total DNA input across all samples, the amount of plasmid DNA was balanced by supplementing with the corresponding empty vector where necessary. Cells were incubated at 37°C for 48 h, and luciferase activity in cell lysates was measured at 560 nm using a microplate reader (Thermo, Waltham, MA, USA).

### Coimmunoprecipitation assays

A coimmunoprecipitation (co-IP) assay was performed as described previously (14). In summary, cells were transfected with the indicated plasmids, and the total amount of DNA was normalized across all samples by supplementing with the corresponding empty vector to ensure equal DNA input. Anti-Flag or anti-HA antibody-conjugated magnetic beads were utilized to capture the immune complexes (10 μL of magnetic beads was combined with 500 μL of whole-cell lysate; MedChemExpress, Monmouth Junction, NJ, USA). Following this, the beads underwent three wash cycles using wash buffer and were then eluted with 1× SDS loading buffer. The resulting samples were analyzed through western blotting.

### Kinetics of virus growth

MDCK/A549 cells were infected with the virus mentioned above (WT or mutant influenza virus) at a multiplicity of infection (MOI) of 0.05. The inoculum was removed after 1 h of virus adsorption. The cells were then washed with PBS and cultured in minimal essential media containing TPCK-treated trypsin. The supernatants of infected cells were collected at 12, 24, and 36 h post-infection (hpi). Viral titers were determined by calculating the TCID_50_ in MDCK cells via the Karber method (15).

### *In vivo* experiments

For the knockdown of KCMF1 *in vivo*, cholesterol-conjugated KCMF1 siRNA or NC siRNA were synthesized for *in vivo* RNA delivery (16). The target sequences for KCMF1 were as follows: 5′-GCCACAGCCAACACAGCCA-3′ (sense) and 5′-UGGCUGUGUUGGCUGUGGC-3′ (antisense). The siRNAs were nasally instilled into 5- to 6-week-old C57/BL6 female specific-pathogen-free mice at days −2, 0, 2, and 4. On day 2, three mice in each group were randomly anesthetized and sacrificed. To detect the knockdown effect of the siRNAs, mouse lungs were homogenized for western blot with an anti-KCMF1 primary antibody. At 6 h after day 0 of treatment, the mice were challenged with 0.5 × MLD50 (1.9 × 10^5^ TCID_50_) of PR8 or mock-infected. The mice were monitored daily for weight loss and mortality over a 14-day period.

To compare the pathogenicity of WT and variant influenza viruses, 6-week-old female C57/BL6 mice were intranasally inoculated with the indicated doses of PR8-WT or PR8-PB1-K653R diluted in PBS (*n* = 10). The mice were monitored daily for weight loss and mortality over a 14-day period. Any mice that lost 20% or more of their initial weight were euthanized humanely. The lungs were harvested on 6 days post-infection (dpi; *n*=3), and the number of viral copies was determined via qRT-PCR.

### Histopathology

The lungs were obtained from individual mice euthanized at various time points, and gross differences in appearance were recorded via digital photography. The lungs were subsequently fixed in 10% neutral-buffered formalin and processed routinely for H&E staining.

### Sensitivity to antiviral compounds *in vitro*

The sensitivity of the PR8-WT or PR8-K653R viruses to baloxavir marboxil (0–200 µM), oseltamivir carboxylate (0–200 µM), or favipiravir (0–200 µM) was evaluated in MDCK cells at an MOI of 0.05. The supernatants were harvested at 48 hpi. Viral titers were determined by calculating the TCID_50_ in MDCK cells. The means and standard errors were plotted, followed by a variable-slope dose-response curve fitting using GraphPad Prism software. A 50% inhibitory concentration (IC50) was extrapolated from the fitted dose-response curve.

### Biosafety statement

All experiments involving the influenza A/Puerto Rico/8/1934 (H1N1) (PR8) virus were conducted under biosafety level 2 (BSL-2) containment in accordance with institutional biosafety guidelines.

### Statistical analysis

Statistical analysis was performed using an unpaired *t*-test or two-way analysis of variance (ANOVA) in GraphPad Prism (version 8.0; GraphPad Software, San Diego, CA, USA). A *P*-value of less than 0.05 was considered significant. The means ± standard errors were shown. ∗, *P* < 0.05; ∗∗, *P* < 0.01; ∗∗∗, *P* < 0.001; ∗∗∗∗, *P* < 0.0001. NS, not statistically significant. The number of mice in each group and specific details on the statistical tests were described in the figure legends. Venn diagrams were generated via a Venn diagram plotter (http://omics.pnl.gov/software/Venn-diagram-plotter).

## RESULTS

### Interaction network between E3 ubiquitin ligases and IAV proteins

**Fig 1:**
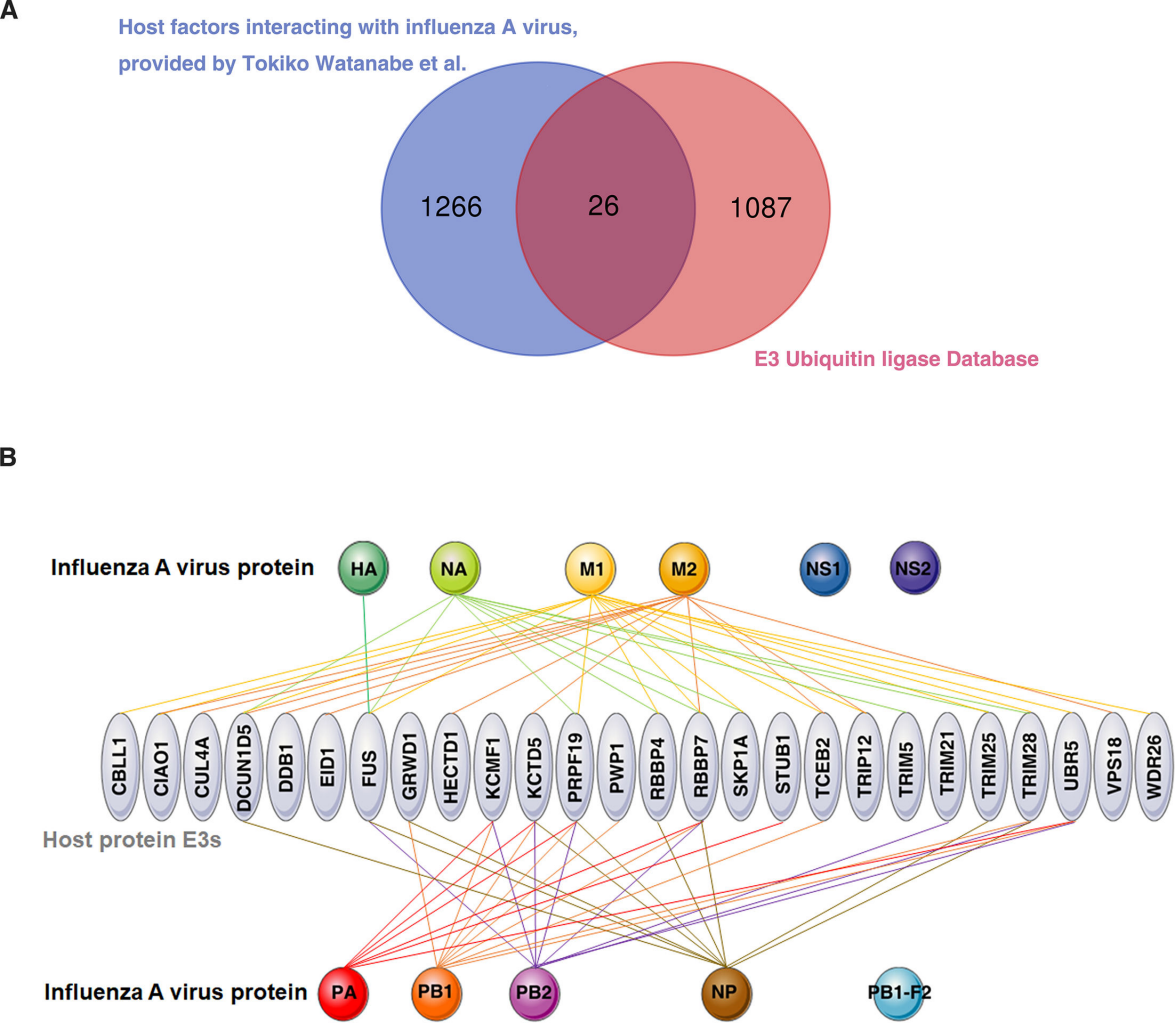
Identification of potential E3 ubiquitin ligases interacting with influenza virus proteins and construction of the interaction network. (**A**) Overlap analysis between the data set reported by Watanabe et al. and an E3 ubiquitin ligase database. (**B**) Interaction network showing 26 candidate E3 ubiquitin ligases and their associations with IAV proteins, including PB2, PB1, PA, HA, NP, NA, M1, M2, NS1, NS2, and PB1-F2.

According to reports by Watanabe and colleagues, a total of 1,292 host proteins were identified to co-precipitate with influenza virus proteins (5, 17). To identify E3 ubiquitin ligases that potentially interact with influenza virus proteins, we further intersected these data with an E3 ligase database. As shown in Fig. 1A, 26 candidate E3 ubiquitin ligases (listed in Table S1) were identified as interacting with influenza virus proteins. Furthermore, the interactions between these E3 ubiquitin ligases and IAV proteins are illustrated in a network diagram (Fig. 1B).

### Validation of candidate genes involved in influenza A virus replication

To systematically evaluate the impact of the 26 candidate host factors on IAV replication, at least three siRNAs targeting each gene were designed, and all sequences are listed in Table S2. siRNAs achieving a knockdown efficiency greater than 65% were selected for subsequent experiments (Fig. S1).

**Fig 2:**
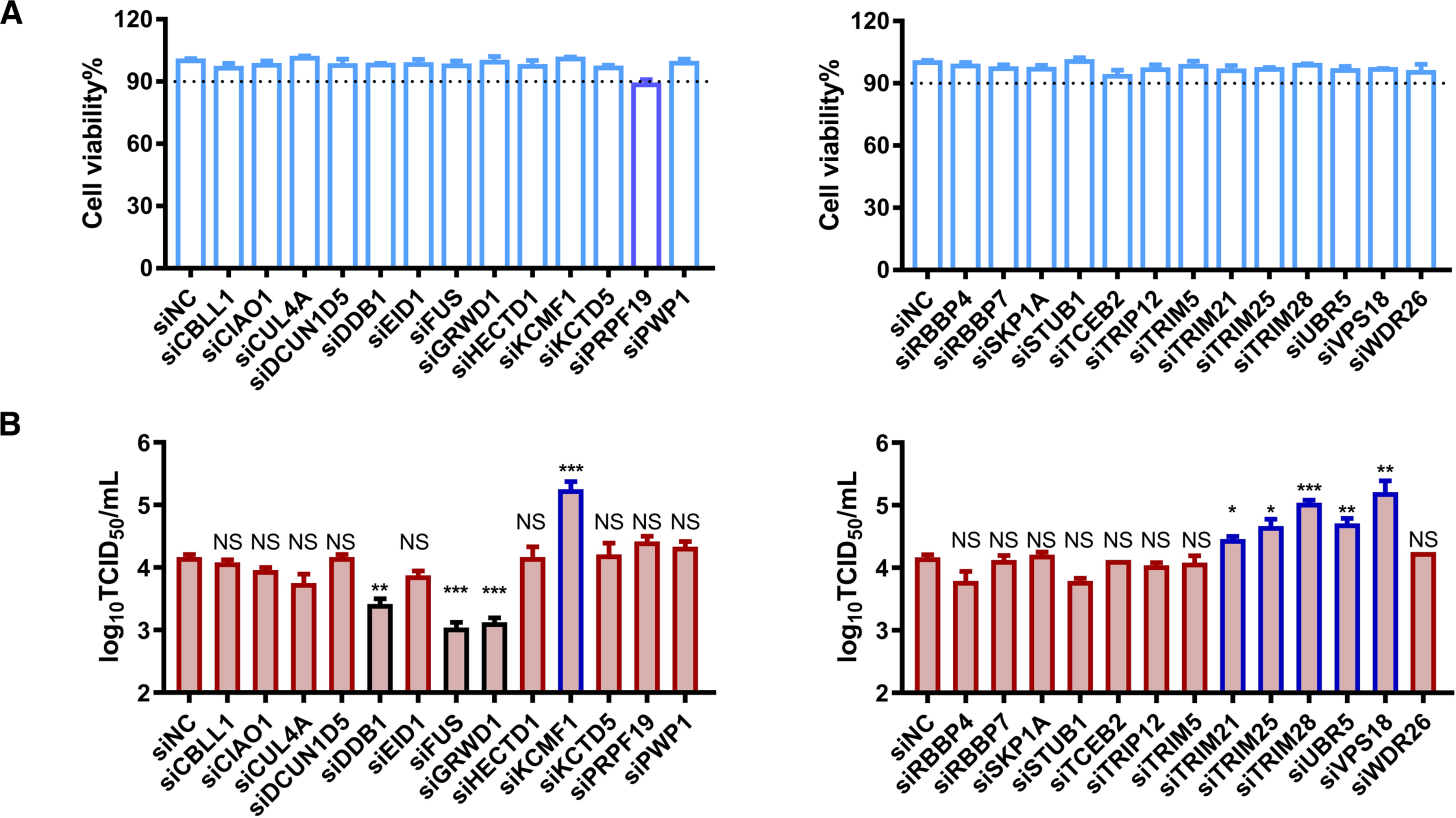
Identification of host factors regulating IAV replication. (**A**) A549 cells were transfected with the indicated siRNAs, and cell viability was assessed by CCK-8 assay at 24 h post-transfection (*n* = 3). (**B**) A549 cells were transfected with the indicated siRNAs and subsequently infected with PR8 virus at 24 h post-transfection. Culture supernatants were collected for TCID_50_ assays. Statistical significance was determined using an unpaired *t*-test. *P < 0.05; **P < 0.01; ***P < 0.001; ****P < 0.0001; NS, not significant.

Prior to assessing their effects on viral replication, we first examined whether siRNA-mediated knockdown of these candidate genes affected cell viability. A CCK-8 assay showed that silencing these genes in A549 cells had no significant impact on cell viability (Fig. 2A).

To further investigate host factors influencing viral replication, A549 cells were transfected with siRNAs targeting each candidate E3 ubiquitin ligase, followed by infection with PR8 (H1N1) at an MOI of 0.05. At 24 h hpi, culture supernatants were collected for TCID_50_ assays, and cell lysates were prepared for western blot analysis. Viral proteins NP and M1, which serve as major structural components of the influenza virus, were used as indicators of viral replication. As shown in Fig. 2B, TCID_50_ assays identified six genes that negatively regulate viral replication and three genes that positively regulate it. Among the negatively regulated genes, KCMF1 exhibited the most pronounced inhibitory effect (Fig. 2B). These results were further supported by western blot analysis of infected cell lysates (Fig. S2). Based on these findings, KCMF1 was selected for further investigation.

### KCMF1 negatively regulates H1N1 influenza virus replication *in vitro* and *in vivo*

**Fig 3:**
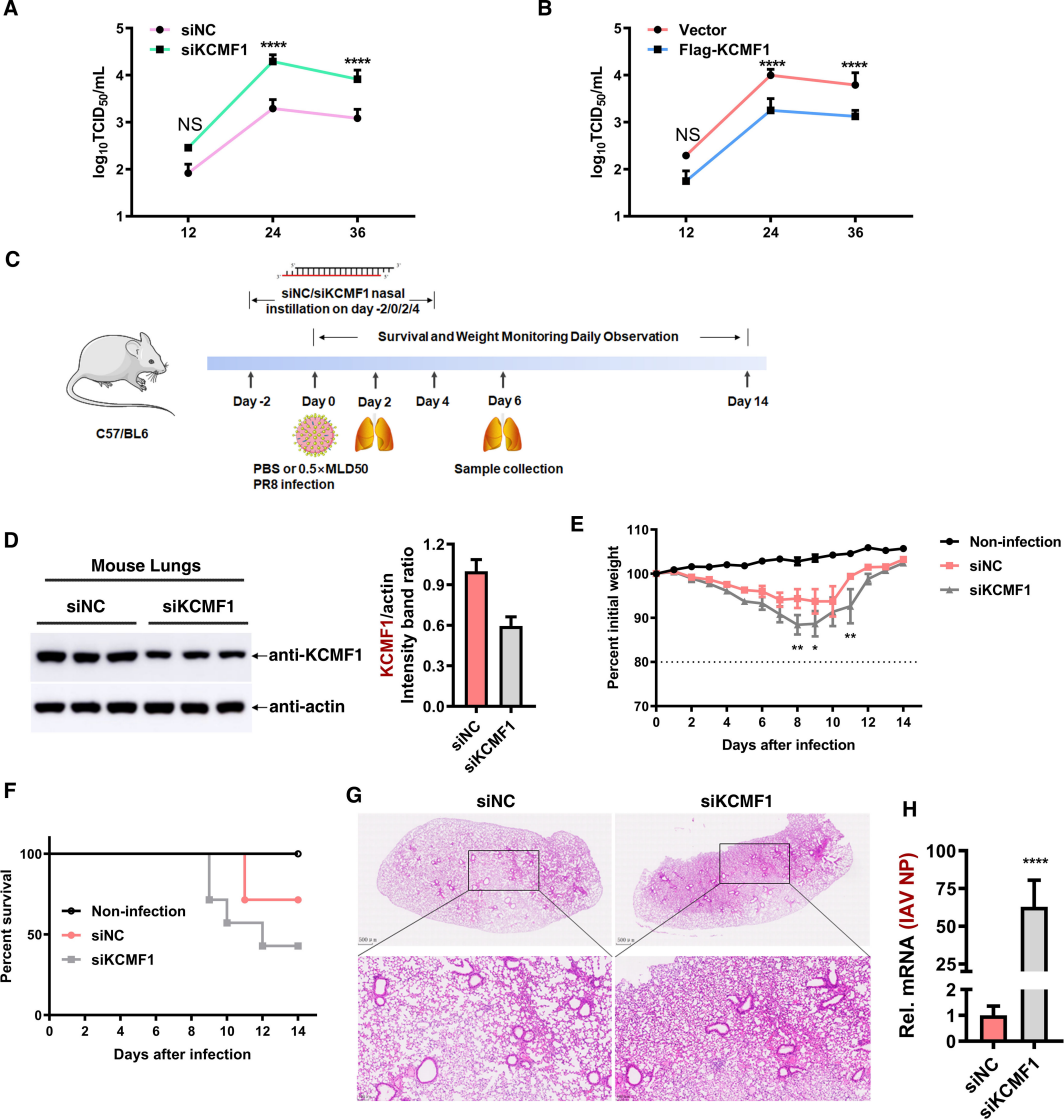
KCMF1 negatively regulates H1N1 influenza virus replication. (**A**) A549 cells were transfected with the indicated siRNAs and infected with PR8 (H1N1) virus at 24 h post-transfection. Culture supernatants were collected at the indicated time points for TCID_50_ assays. (**B**) A549 cells were transfected with the indicated plasmids and subsequently infected with PR8 (H1N1) virus at 24 h post-transfection. Culture supernatants were collected at the indicated time points for TCID_50_ assays. (**C**) Schematic representation of the experimental timeline for *in vivo* studies. (**D**) KCMF1 knockdown efficiency in mouse lungs was assessed by western blot analysis. (**E and F**) Five- to 6-week-old female C57BL/6 specific-pathogen-free (SPF) mice were intranasally infected with 0.5 × MLD_50_ (1.9 × 10^5^ TCID_50_) of PR8 virus. Body weight (**E**) and survival (**F**) were monitored daily for 14 days (*n* = 7). (**G**) Mouse lungs were collected at 6 days post-infection (dpi), and lung sections were subjected to hematoxylin and eosin (H&E) staining. Scale bar, 500 μm. (**H**) Viral RNA copy numbers in mouse lungs at 6 dpi were quantified by qRT-PCR. Data are presented as means ± SD from three independent experiments. Statistical significance was determined using two-way ANOVA (**A, B, and E**) or an unpaired *t*-test (**H**). *P < 0.05; **P < 0.01; ***P < 0.001; ****P < 0.0001; NS, not significant.

To further substantiate the regulatory role of KCMF1 in influenza virus infection, we assessed its impact on viral replication at multiple time points post-infection. Culture supernatants were collected at 12, 24, and 36 hpi for TCID_50_ assays. Knockdown of KCMF1 using siRNA (siKCMF1) resulted in a marked increase in viral titers at 24 and 36 hpi compared with cells transfected with negative control siRNA (siNC; Fig. 3A). Consistently, viral protein levels were significantly elevated in siKCMF1-treated cells relative to siNC controls (Fig. S3A).

We next examined the effect of KCMF1 overexpression on viral replication in A549 cells. A Flag-tagged KCMF1 expression plasmid was transfected into A549 cells, followed by infection with PR8 (H1N1) at an MOI of 0.05 at 24 h post-transfection (hpt). Viral titers in the culture supernatants were measured at the indicated time points. As expected, KCMF1 overexpression significantly reduced viral titers (Fig. 3B) and decreased viral protein levels in infected cells (Fig. S3B).

Moreover, to investigate the role of KCMF1 in influenza virus infection *in vivo*, we initially attempted to generate KCMF1^−/−^ double knockout mice; however, these mice exhibited embryonic lethality, precluding their successful establishment. Therefore, an siRNA-based delivery system was employed to achieve KCMF1 knockdown *in vivo* (Fig. 3C). The KCMF1-targeting siRNA achieved an approximate knockdown efficiency of 43.2% in mouse lungs (Fig. 3D). Mice were intranasally challenged with 0.5 × MLD₅₀ (1.9 × 10^5^ TCID_50_) of PR8 virus or mock-treated on days 0, 2, and 4 following the initial siRNA administration. Notably, mice in the siKCMF1-treated group exhibited significantly greater body weight loss compared with those in the siNC group (Fig. 3E). In addition, KCMF1 knockdown shortened survival by approximately 2 days and increased mortality by 28.6% (Fig. 3F). Furthermore, siKCMF1-treated mice displayed more severe lung pathology and higher viral loads than siNC-treated controls (Fig. 3G and H). To investigate the role of KCMF1 *in vivo*, we initially attempted to generate KCMF1^−/−^ knockout mice; however, embryonic lethality precluded their establishment. Therefore, an siRNA-based delivery approach was used to achieve KCMF1 knockdown *in vivo* (Fig. 3C). KCMF1-targeting siRNA achieved an approximate knockdown efficiency of 43.2% in mouse lungs (Fig. 3D). Mice were intranasally challenged with 0.5 × MLD_50_ (1.9 × 10⁵ TCID_50_) of PR8 (H1N1) virus on days 0, 2, and 4 following the initial siRNA administration. Body weight and survival were monitored daily for 14 dpi. Notably, mice treated with siKCMF1 exhibited significantly greater body weight loss compared with siNC-treated controls (Fig. 3E). In addition, KCMF1 knockdown shortened survival by approximately 2 days and increased mortality by 28.6% (Fig. 3F). Furthermore, siKCMF1-treated mice displayed more severe lung pathology and higher viral loads than control mice (Fig. 3G and H).

Collectively, these results indicate that KCMF1 negatively regulates H1N1 IAV replication *in vitro* and *in vivo*.

### KCMF1 interacts with PB1

**Fig 4:**
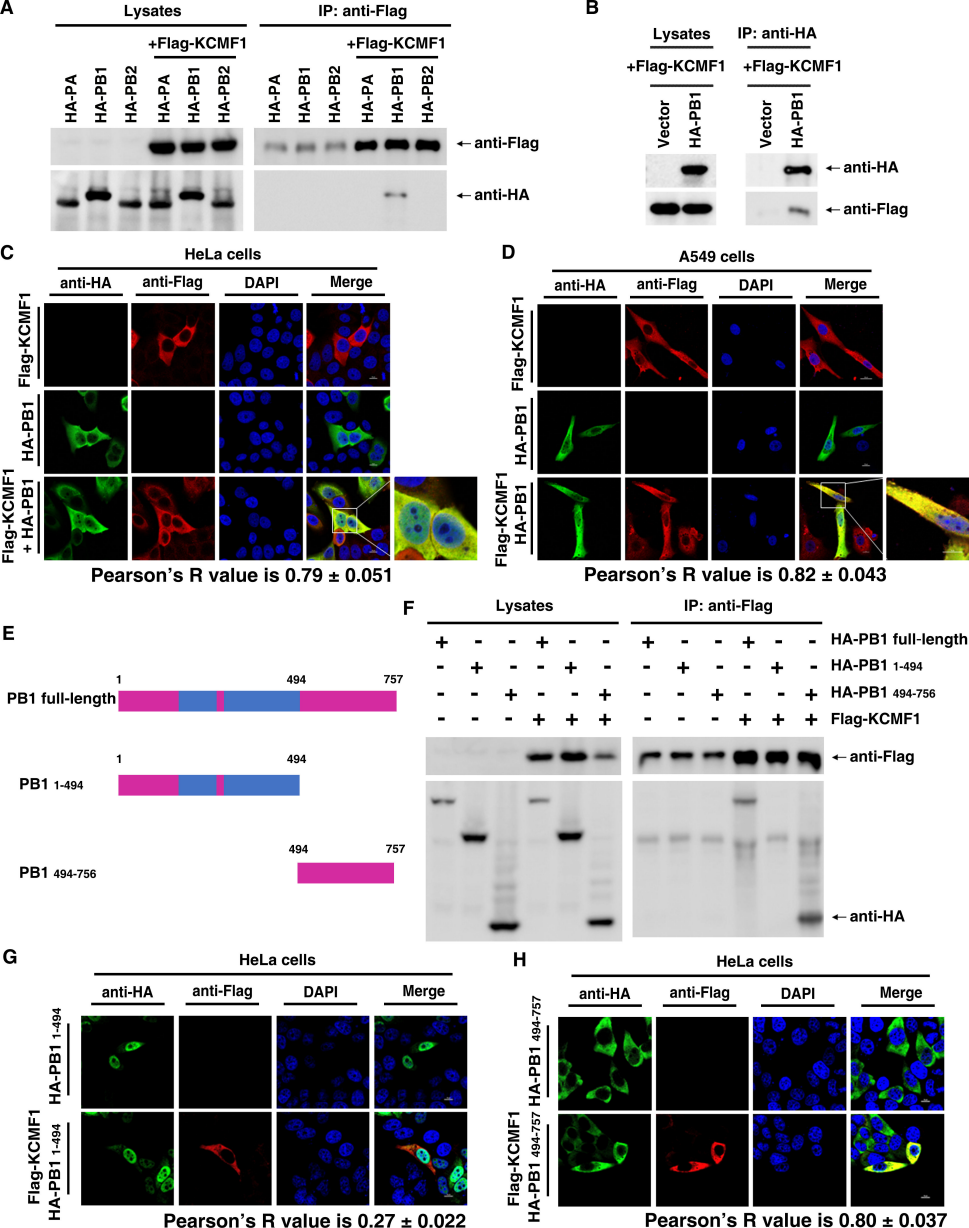
Interaction between KCMF1 and PB1. (**A**) HEK293T cells were co-transfected with plasmids encoding HA-PA, HA-PB1, HA-PB2, and Flag-KCMF1. Cell lysates were subjected to anti-Flag immunoprecipitation, followed by western blot analysis. (**B**) HEK293T cells were co-transfected with HA-PB1 and Flag-KCMF1 plasmids. Cell lysates were subjected to anti-HA immunoprecipitation and analyzed by western blot. (**C and D**) HeLa (**C**) and A549 (**D**) cells were co-transfected with Flag-KCMF1 and HA-PB1 plasmids, and colocalization was assessed by confocal microscopy. (**E**) Schematic representation of the IAV PB1 protein, indicating the N-terminal (1–494) and C-terminal (494–757) regions. (**F**) HEK293T cells were transfected with plasmids encoding full-length HA-PB1, HA-PB1 (1–494), HA-PB1 (494–757), and Flag-KCMF1. Cell lysates were subjected to anti-Flag immunoprecipitation and analyzed by western blot. (**G and H**) HeLa cells were co-transfected with Flag-KCMF1 and truncated PB1 constructs [HA-PB1 (1–494) or HA-PB1 (494–757)]. Colocalization of HA-PB1 (1–494) (**G**) or HA-PB1 (494–757) (**H**) with Flag-KCMF1 was analyzed by confocal microscopy. Scale bars, 10 μm. Colocalization was quantified using Pearson’s correlation coefficient (*R*) with ImageJ (*n* = 10).

Based on the interaction network shown in Fig. 1B, KCMF1 was predicted to interact with the IAV polymerase subunits PB1, PB2, and PA. To validate these interactions, co-IP assays were performed. As shown in Fig. 4A, Flag-tagged KCMF1 physically interacted with HA-tagged PB1 in HEK293T cells, which was further confirmed by reciprocal co-IP experiments (Fig. 4B). In addition, confocal microscopy analysis revealed that HA-PB1 colocalized with Flag-KCMF1 in both HeLa cells (Pearson’s correlation coefficient, *R* = 0.79 ± 0.051) and A549 cells (*R* = 0.82 ± 0.043; Fig. 4C and D). To further examine this interaction under viral infection conditions, A549 cells transfected with Flag-KCMF1 were subsequently infected with PR8 virus. Consistently, confocal microscopy demonstrated colocalization of Flag-KCMF1 and PB1 in PR8-infected cells (*R* = 0.64 ± 0.041; Fig. S4).

We next mapped the PB1 regions responsible for interaction with KCMF1. Based on previous structural studies (11, 18), PB1 was divided into two fragments: the N-terminal region (PB1_1–494_) and the C-terminal region (PB1_494–757_; Fig. 4E). Co-IP assays showed that the C-terminal fragment (HA-PB1_494–757_) interacted with Flag-KCMF1 (Fig. 4F). Consistently, confocal microscopy analysis revealed limited colocalization between Flag-KCMF1 and the N-terminal fragment (PB1_1–494_; *R* = 0.27 ± 0.022; Fig. 4G), whereas markedly stronger colocalization was observed with the C-terminal fragment (PB1_494–757_; *R* = 0.80 ± 0.037; Fig. 4H).

### KCMF1-mediated ubiquitination of PB1 promotes its proteasomal degradation

**Fig 5:**
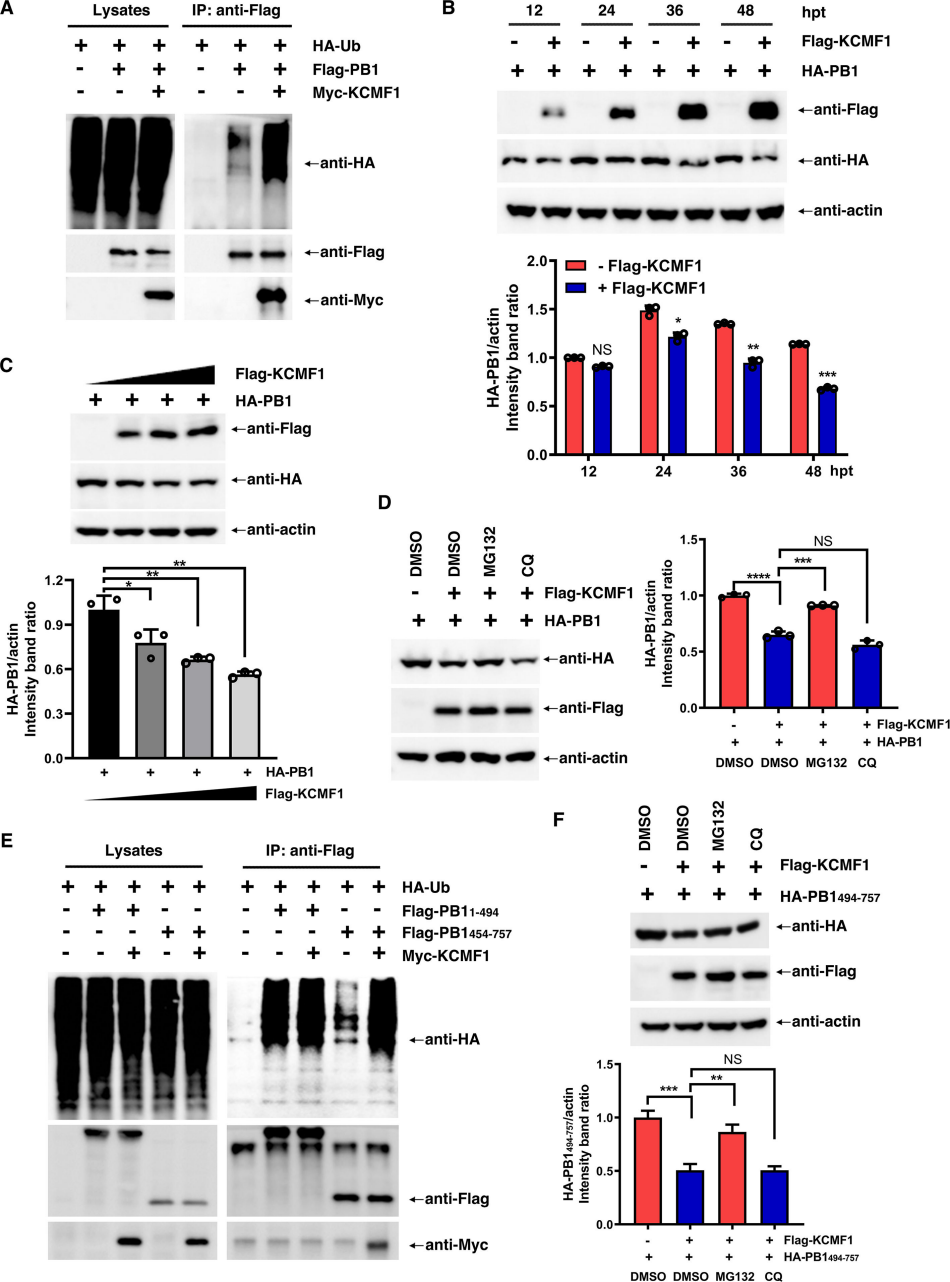
KCMF1-mediated ubiquitination of PB1 and its association with proteasomal degradation. (**A**) HEK293T cells overexpressing KCMF1 were transfected with the indicated plasmid combinations to assess PB1 ubiquitination. (**B**) HEK293T cells were co-transfected with Flag-KCMF1 and HA-PB1 plasmids and harvested at the indicated time points. Cell lysates were analyzed by western blot. (**C**) HEK293T cells overexpressing KCMF1 were co-transfected with increasing amounts of HA-PB1 plasmid (0, 0.25, 0.5, or 1.0 μg per well in 12-well plates). Total plasmid amounts were equalized using an empty vector. Cells were collected at 24 hpt for protein analysis. (**D**) HEK293T cells were co-transfected with Flag-KCMF1 and HA-PB1. At 24 h post-transfection, cells were treated with DMSO, MG132, or chloroquine (CQ) for 12 h, followed by western blot analysis. (**E**) HEK293T cells overexpressing KCMF1 were transfected with the indicated plasmid combinations to assess ubiquitination of PB1 (1–494) and PB1 (494–757) fragments. (**F**) HEK293T cells were co-transfected with Flag-KCMF1 and HA-PB1 (1–494) or HA-PB1 (494–757). At 24 hpt, cells were treated with DMSO, MG132, or CQ for 12 h, and cell lysates were analyzed by western blot. Data are presented as means ± SD from three independent experiments. Statistical significance was determined using two-way ANOVA (**B**) or an unpaired *t*-test (**C, D, and F**). *P < 0.05; **P < 0.01; ***P < 0.001; ****P < 0.0001; NS, not significant.

Given the interaction between KCMF1 and PB1, together with the reported E3 ubiquitin ligase activity of KCMF1, we hypothesized that KCMF1 mediates the ubiquitination of PB1. To test this, HEK293T cells were co-transfected with plasmids encoding Flag-PB1, HA-ubiquitin (HA-Ub), and Myc-KCMF1. The results showed that PB1 underwent ubiquitination, which was markedly enhanced upon KCMF1 overexpression (Fig. 5A).

Ubiquitination is a critical post-translational modification that can target proteins for proteasomal degradation or regulate their function (19). Given that KCMF1 negatively regulates influenza virus replication, we next investigated whether it promotes PB1 degradation. HEK293T cells were co-transfected with Flag-KCMF1 and HA-PB1 plasmids and harvested at the indicated time points. As shown in Fig. 5B, PB1 protein levels were reduced at 24 h post-transfection in the presence of KCMF1. Moreover, PB1 degradation increased in a dose-dependent manner with increasing levels of Flag-KCMF1 expression (Fig. 5C).

To further elucidate the mechanism underlying KCMF1-mediated PB1 degradation, we employed chemical inhibitors targeting distinct protein degradation pathways. Treatment with the proteasome inhibitor MG132 restored PB1 protein levels, indicating that PB1 degradation is mediated through the ubiquitin-proteasome pathway. In contrast, chloroquine, an inhibitor of lysosomal acidification that disrupts autophagy-lysosome-mediated degradation, had no significant effect on PB1 abundance. These findings suggest that KCMF1 primarily promotes PB1 degradation via the proteasome rather than the lysosomal pathway (Fig. 5D).

We next mapped the ubiquitination region within PB1 and found that KCMF1 specifically mediated ubiquitination of the C-terminal domain (amino acids 494–757), whereas the N-terminal domain (amino acids 1–494) was not affected (Fig. 5E). Consistent with this, KCMF1 selectively promoted the degradation of the PB1 (494–757) fragment via the ubiquitin–proteasome pathway (Fig. 5F).

Collectively, these results indicate that KCMF1 targets the C-terminal region (494–757) of PB1 for ubiquitination and is associated with its proteasomal degradation.

### The K653 residue of PB1 is critical for IAV polymerase activity and KCMF1-mediated ubiquitination

**Fig 6:**
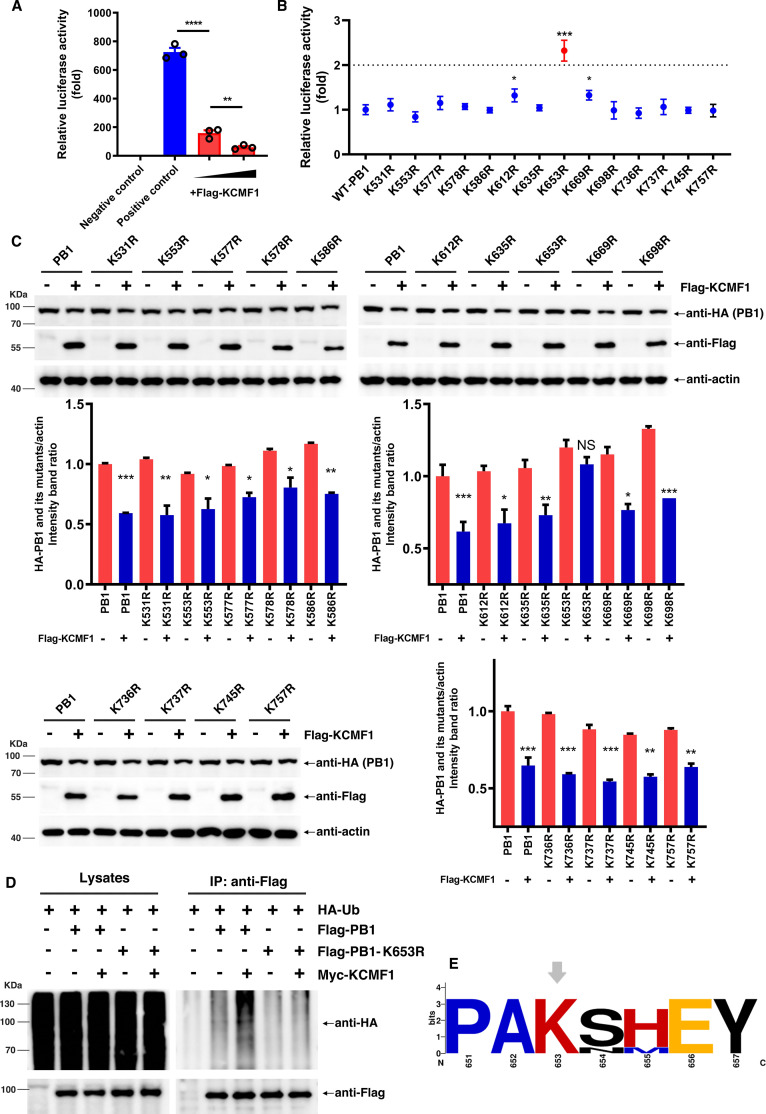
The PB1 K653 residue is important for IAV polymerase activity and KCMF1-mediated ubiquitination. (**A**) HEK293T cells were co-transfected with Flag-KCMF1 and influenza virus polymerase reporter plasmids, including a Pol I-driven reporter plasmid together with PB1, PB2, PA, and NP expression plasmids. At 36 hpt, cells were harvested for dual-luciferase reporter assays. The negative control lacked the PA plasmid, whereas the positive control included the complete polymerase complex. (**B**) HEK293T cells were co-transfected with Flag-KCMF1 and polymerase reporter plasmids containing different PB1 mutants. At 36 hpt, polymerase activity was assessed by dual-luciferase reporter assays. (**C**) HEK293T cells were co-transfected with Flag-KCMF1 or empty vector together with the indicated PB1 mutants. At 36 hpt, cell lysates were analyzed by western blot. (**D**) HEK293T cells overexpressing Flag-PB1 were transfected with the indicated plasmid combinations to assess ubiquitination of wild-type PB1 and the K653 mutant. (**E**) Sequence logo analysis of PB1 amino acid sequences from multiple IAV subtypes (H1N1, H2N2, H3N2, H5N1, H5N6, H7N9, and H9N2), generated using WebLogo. Data are presented as means ± SD from three independent experiments. Statistical significance was determined using an unpaired *t*-test (**A and B**) or two-way ANOVA (**C**). *P < 0.05; **P < 0.01; ***P < 0.001; ****P < 0.0001; NS, not significant.

These findings demonstrate that KCMF1 promotes the ubiquitination and degradation of PB1. As PB1 serves as the core catalytic subunit of the influenza virus polymerase complex, we next investigated whether KCMF1 affects viral polymerase activity. As expected, KCMF1 overexpression markedly suppressed influenza virus polymerase activity (Fig. 6A). Based on these observations, we hypothesized that mutation of a key ubiquitination site on PB1 targeted by KCMF1 may impair the regulatory effect of KCMF1 on IAV polymerase activity.

To further investigate this possibility, all 14 lysine residues within the PB1 C-terminal region (amino acids 494–757; K531, K553, K577, K578, K586, K612, K635, K653, K669, K698, K736, K737, K745, and K757) were individually mutated to arginine by site-directed mutagenesis. Among these mutants, PB1-K653R exhibited the highest polymerase activity in the presence of PA, PB2, and NP under conditions of KCMF1 overexpression (Fig. 6B), suggesting that K653 plays an important role in KCMF1-mediated regulation of PB1.

To further validate this observation, each PB1 mutant was co-transfected with either KCMF1 or an empty vector into HEK293T cells, followed by analysis of PB1 protein levels. Western blot analysis showed that the mutation at K653 markedly attenuated KCMF1-associated PB1 degradation (Fig. 6C). Consistently, ubiquitination assays revealed that the K653R mutation significantly reduced KCMF1-mediated ubiquitination of PB1 (Fig. 6D).

Together, these findings identify K653 as a key residue involved in KCMF1-associated ubiquitination and regulation of IAV PB1. Furthermore, sequence logo analysis of IAV PB1 generated using WebLogo 3 (20) demonstrated that the K653 residue is highly conserved (Fig. 6E).

### PB1 K653 mutation promotes viral replication and impairs KCMF1-mediated antiviral activity

**Fig 7:**
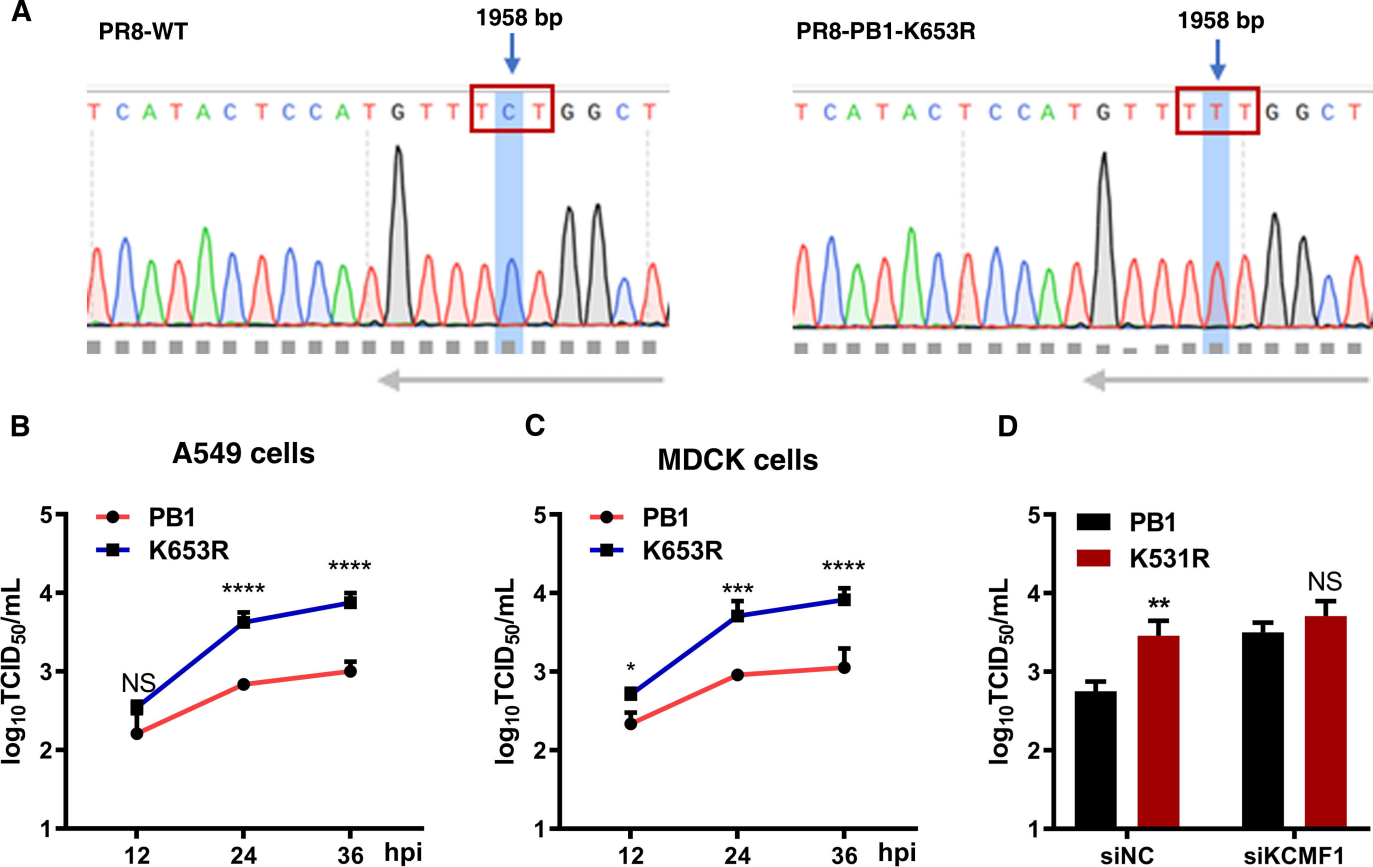
Effects of the PB1 K653 mutation on viral replication *in vitro*. (**A**) The PB1 gene sequences of PR8-PB1-WT and PR8-PB1-K653R viruses were confirmed by Sanger sequencing. (**B and C**) A549 (**B**) and MDCK (**C**) cells were infected with PR8-PB1-WT or PR8-PB1-K653R at an MOI of 0.05. Culture supernatants were collected at the indicated time points for viral titration by TCID_50_ assay. (**D**) A549 cells were transfected with the indicated siRNAs and subsequently infected with PR8-PB1-WT or PR8-PB1-K653R at an MOI of 0.05. Culture supernatants were collected for TCID_50_ analysis. Data are presented as means ± SD from three independent experiments. Statistical significance was determined using two-way ANOVA. *P < 0.05; **P < 0.01; ***P < 0.001; ****P < 0.0001; NS, not significant.

To assess the impact of the PB1 K653 mutation on the influenza virus life cycle, wild-type PR8 (PR8-WT) and mutant PR8-PB1-K653R (abbreviated as K653R in the figures) viruses were generated. The mutation was confirmed by sequencing analysis (Fig. 7A). Viral replication kinetics were evaluated in MDCK and A549 cells infected with PR8-WT or PR8-PB1-K653R at an MOI of 0.05. Cell lysates and culture supernatants were collected at 12, 24, and 36 hpi for analysis. Viral titration showed that the K653R mutation markedly increased viral yields in the supernatants (Fig. 7B and C). Consistently, immunoblot analysis revealed that the levels of viral NP and M1 proteins were significantly elevated in cells infected with the PB1-K653R mutant compared with those infected with PR8-WT (Fig. S5A and B). These results indicate that the PB1 K653 mutation enhances influenza virus replication *in vitro*.

Given that K653 represents a key site for KCMF1-associated ubiquitination of PB1, we next investigated whether the antiviral effect of KCMF1 depends on this residue. A549 cells with or without KCMF1 knockdown were infected with PR8-WT or PR8-PB1-K653R. At 24 hpi, culture supernatants were collected for TCID_50_ assays, followed by western blot analysis of cell lysates. KCMF1 knockdown significantly increased the viral titers of PR8-WT. In contrast, KCMF1 knockdown had no significant effect on the viral titers of PR8-PB1-K653R (Fig. 7D). Consistent with these findings, viral protein levels in cell lysates showed a similar trend (Fig. S5C).

Collectively, these results indicate that the PB1 K653 residue is an important determinant of KCMF1-associated restriction of influenza virus replication, highlighting its role in the antiviral function of KCMF1 against PR8 (H1N1).

### PB1 K653R mutation enhances the pathogenicity of PR8 influenza virus in mice

**Fig 8:**
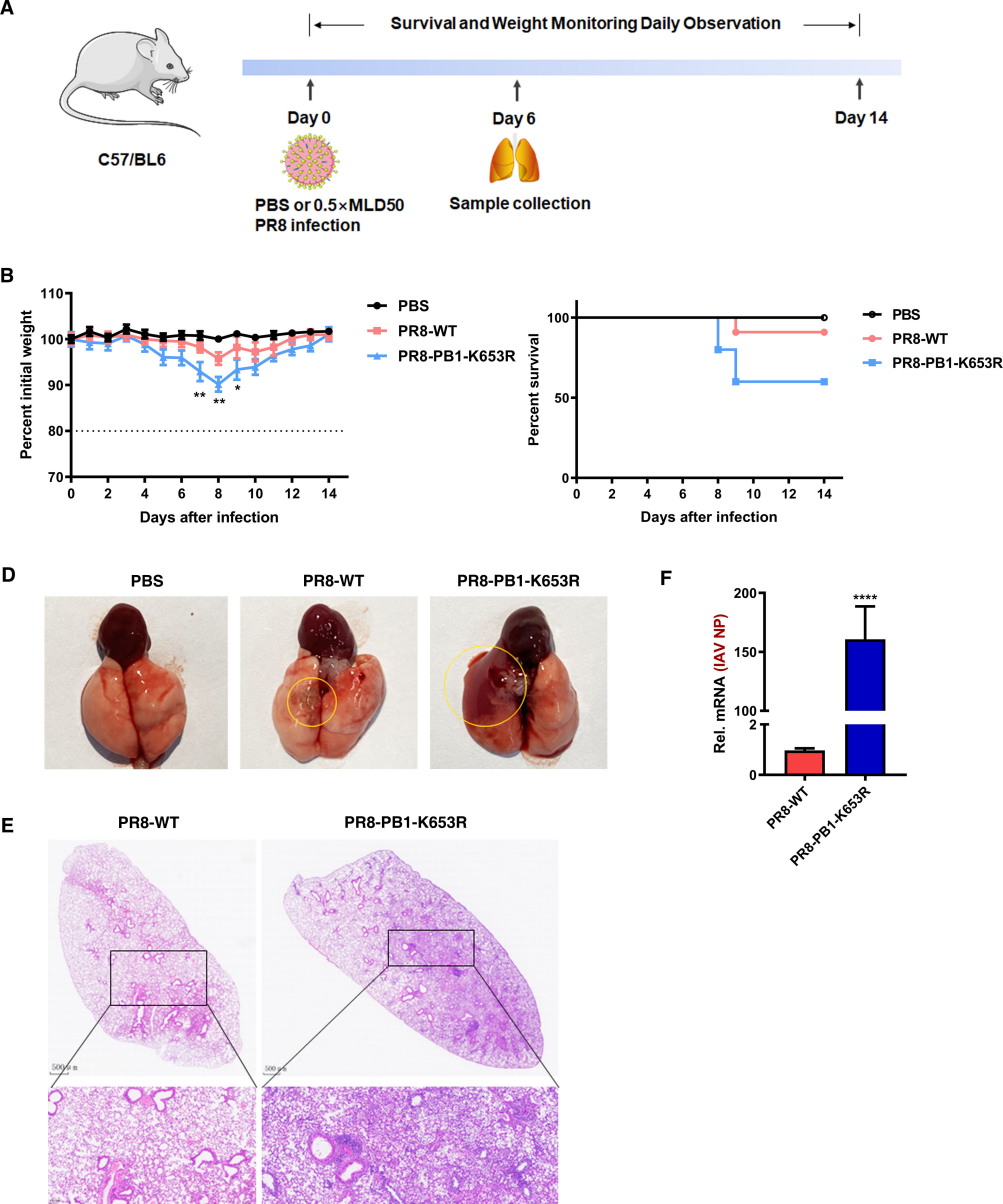
PB1 K653 mutation enhances the pathogenicity of PR8 (H1N1) influenza virus in mice. (**A**) Six-week-old C57BL/6J mice were intranasally infected with 0.5 × MLD_50_ (1.9 × 10^5^ TCID₅₀) of PR8-WT or PR8-PB1-K653R virus. Mice were euthanized at 6 dpi for tissue collection. (**B and C**) Body weight loss (**B**) and survival (**C**) were monitored daily for 14 days (*n* = 10 per group). (**D**) Representative gross morphology of lung tissues at 6 dpi. Focal dark red lesions are indicated (circled). (**E**) Lung tissues were collected at 6 dpi and subjected to histopathological analysis by H&E staining. Scale bar, 500 μm. (**F**) Viral RNA copy numbers in lung tissues at 6 dpi were quantified by qRT-PCR. Data are presented as means ± SD from three independent experiments. Statistical significance was determined using two-way ANOVA (**B**) or an unpaired *t*-test (**F**). *P < 0.05; **P < 0.01; ***P < 0.001; ****P < 0.0001; NS, not significant.

Given that the PB1 K653R mutation enhances viral replication *in vitro*, we next evaluated its impact on viral pathogenicity *in vivo*. Six-week-old C57BL/6 mice were intranasally inoculated with PR8-WT or PR8-PB1-K653R virus at a dose of 0.5 × MLD₅₀ (1.9 × 10^5^ TCID_50_), as described previously (1) (Fig. 8A). Over a 14-day observation period, mice infected with PR8-PB1-K653R exhibited greater body weight loss and more severe clinical symptoms compared with those infected with PR8-WT. Notably, the mortality rate reached 40% in the PR8-PB1-K653R group, whereas only 10% mortality was observed in the PR8-WT group (Fig. 8B and C).

Gross pathological examination revealed that lungs from the uninfected control group (PBS-treated) appeared normal, with a pinkish coloration. In contrast, mice infected with PR8-PB1-K653R exhibited more extensive lung lesions than those infected with PR8-WT at 6 dpi (Fig. 8D). Histopathological analysis further showed that PR8-PB1-K653R infection resulted in more severe tissue damage and increased inflammatory cell infiltration compared with PR8-WT infection (Fig. 8E). Consistently, mice infected with the PB1-K653R mutant virus displayed significantly higher viral loads in the lungs than those infected with PR8-WT, with viral RNA copy numbers increased by more than 150-fold (Fig. 8F).

Collectively, these results indicate that the PB1 K653R mutation markedly enhances the virulence of PR8 (H1N1) influenza virus in mice.

### The PB1 K653R mutation confers reduced sensitivity to favipiravir

Given that the PB1 K653R mutation enhances influenza virus replication and virulence both *in vitro* and *in vivo*, we next evaluated its sensitivity to commonly used antiviral agents. To this end, we assessed the susceptibility of PR8-WT and PR8-PB1-K653R viruses to three anti-influenza drugs using an enzyme-based neuraminidase inhibition assay. The drugs tested included oseltamivir (a neuraminidase inhibitor), baloxavir marboxil (a cap-dependent endonuclease inhibitor), and favipiravir (a viral RNA polymerase inhibitor).

**Fig 9:**
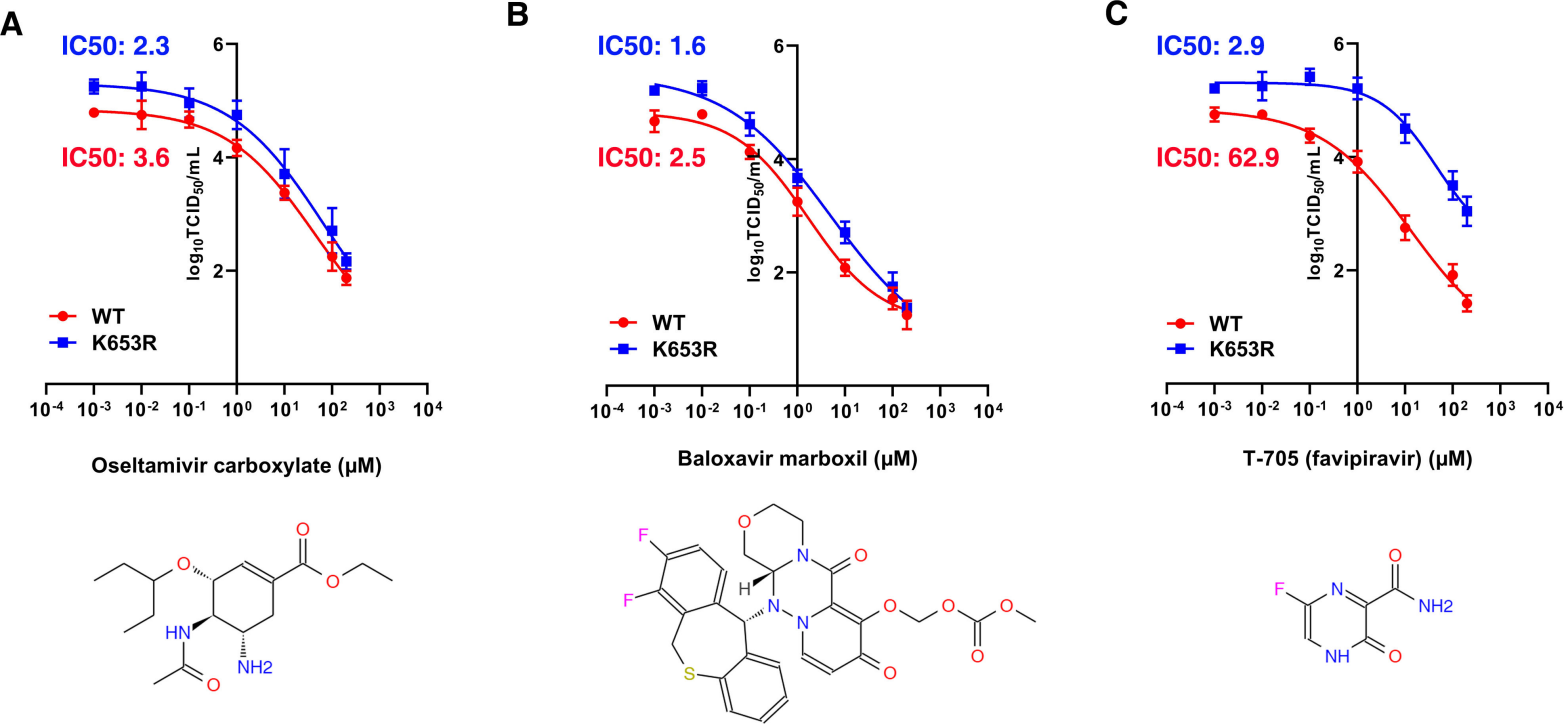
Sensitivity of PR8-WT and PR8-PB1-K653R influenza viruses to antiviral agents *in vitro*. Dose-response curves for oseltamivir (**A**), baloxavir marboxil (**B**), and favipiravir (T-705) (**C**) were generated in MDCK cells infected with recombinant PR8-WT or PR8-PB1-K653R viruses. MDCK cells were infected at an MOI of 0.05, and supernatants were collected at 48 hpi for viral titration. Viral titers are presented as log_10_ TCID_50_/mL (mean ± SD) from three independent replicates.

The IC50 values for oseltamivir and baloxavir marboxil were comparable between PR8-WT and PR8-PB1-K653R (Fig. 9A and B). In contrast, the PR8-PB1-K653R mutant exhibited markedly reduced sensitivity to favipiravir, with an IC50 of 62.9 μM compared with 2.9 μM for PR8-WT (Fig. 9C).

## DISCUSSION

Post-translational modifications, including phosphorylation, acetylation, SUMOylation, and ubiquitination, are critical regulators of protein activity, localization, and stability (21). Among these, ubiquitination plays a central role in modulating protein turnover and function. Multiple IAV proteins, including M1, M2, PB2, and NP, have been reported to undergo ubiquitination in both transfected and infected cells (1, 22–24). Increasing evidence further indicates that site-specific ubiquitination of viral proteins can profoundly influence the viral life cycle. As the catalytic core of the IAV RNA polymerase complex, PB1 is essential for viral transcription and replication and is subject to regulation by the ubiquitin–proteasome system (25). For instance, the E3 ubiquitin ligase TRIM32 has been shown to interact with PB1 and promote its ubiquitination (26). However, the molecular determinants and functional consequences of PB1 ubiquitination remain incompletely defined.

**Fig 10:**
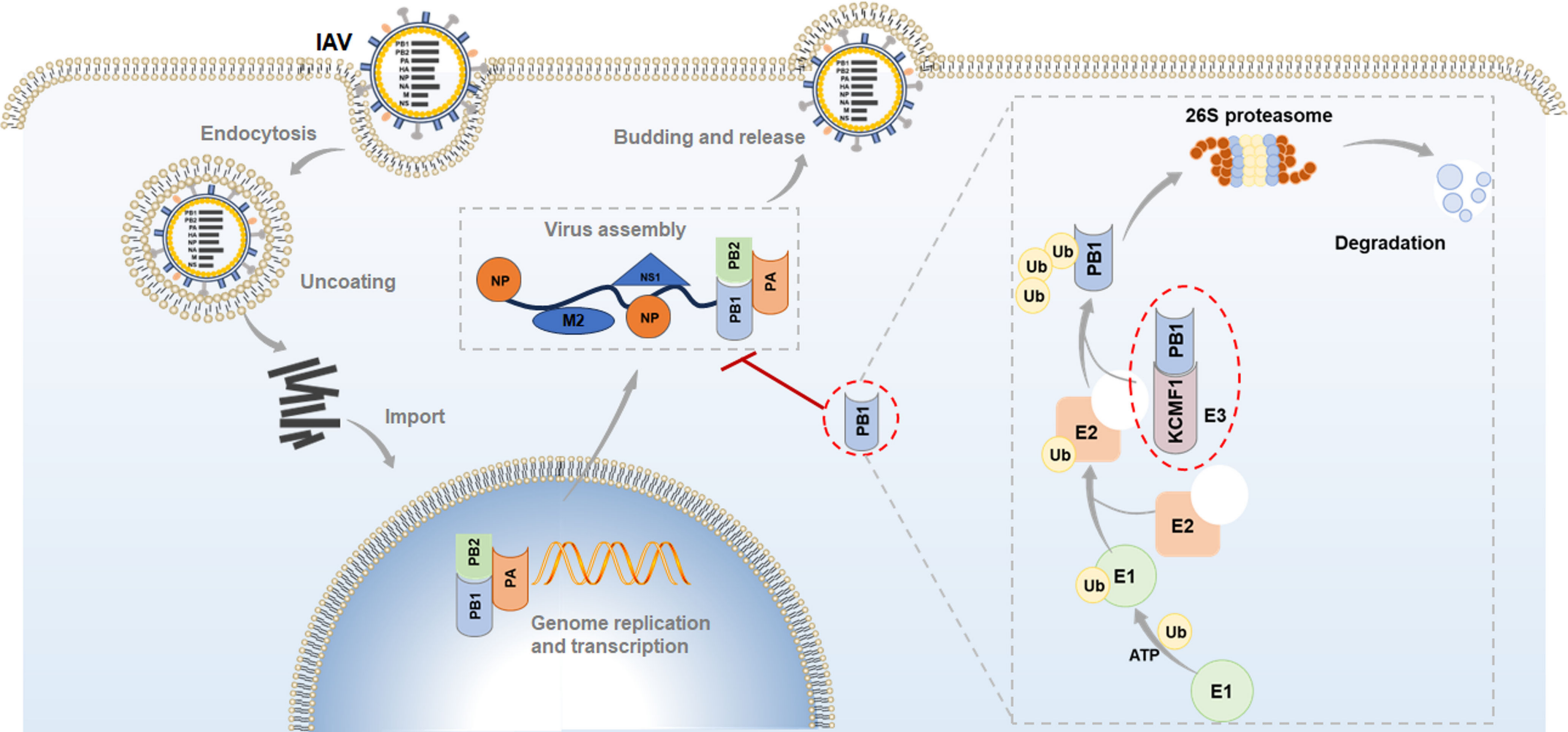
Schematic illustration of the mechanism by which KCMF1 regulates IAV replication. KCMF1 interacts with the influenza virus PB1 protein and mediates its ubiquitination, which is associated with PB1 degradation via the ubiquitin–proteasome pathway.

In this study, we identify the E3 ubiquitin ligase KCMF1 as a previously unrecognized host factor that interacts with PB1 and restricts IAV replication. We demonstrate that KCMF1 mediates PB1 ubiquitination and promotes its degradation via the proteasome pathway. Importantly, we map K653 as a critical ubiquitination site within the C-terminal region of PB1. Substitution of this residue (K653R) markedly attenuates KCMF1-mediated ubiquitination and degradation of PB1, thereby diminishing the inhibitory effect of KCMF1 on viral polymerase activity. Consistent with this, the K653R mutation enhances viral replication *in vitro* and increases pathogenicity *in vivo*, highlighting K653 as a key regulatory node in host-mediated restriction of IAV replication (Fig. 10).

Although the PB1 K653 residue is highly conserved across multiple influenza virus subtypes, the emergence of mutations at this site during long-term host-virus interactions and viral evolution cannot be excluded. In this study, the PB1-K653R mutation markedly enhances viral replication both *in vitro* and *in vivo*, while reducing sensitivity to the polymerase inhibitor favipiravir. Accordingly, such mutations, if they arise, may have important implications for viral pathogenicity and antiviral susceptibility. These findings may provide a foundation for the development of future antiviral strategies and surveillance efforts.

From a structural perspective, the K653 residue is positioned within the C-terminal region of PB1, a domain implicated in polymerase assembly and interactions with other components of the viral ribonucleoprotein complex. Previous studies have shown that this region contributes to the stabilization of the polymerase complex and mediates interactions with PB2 and PA. Therefore, in addition to regulating PB1 stability through KCMF1-mediated ubiquitination at this site, the K653R mutation may alter the local structural context or perturb critical interaction interfaces, thereby enhancing polymerase activity and viral replication. Although the precise structural consequences of this substitution remain to be determined, our findings support a model in which K653 serves as a functionally important residue linking posttranslational modification to the regulation of polymerase activity.

KCMF1 has previously been characterized as a potassium channel modulatory factor and has also been implicated in ubiquitin-dependent regulatory pathways (27). More recently, its potential role as an E3 ubiquitin ligase has attracted increasing attention (28–30). Notably, KCMF1 has been reported to mediate K63-linked ubiquitination (31), a modification typically associated with signaling functions rather than proteasomal degradation. In the present study, we demonstrate that KCMF1 promotes the ubiquitination of PB1 and facilitates its degradation. Although the specific ubiquitin linkage type was not determined, the observed degradation suggests that KCMF1 may regulate PB1 stability through alternative ubiquitin chain linkages.

In addition to its central role in viral RNA transcription and replication, PB1 has also been implicated in immune evasion. Previous studies have shown that PB1 from H7N9 influenza virus suppresses type I interferon signaling by promoting mitochondrial antiviral-signaling protein (MAVS) degradation through an autophagy-dependent mechanism involving RNF5 and the selective autophagy receptor NBR1 (32). In contrast, our findings identify a distinct regulatory pathway in which the host E3 ubiquitin ligase KCMF1 targets PB1 itself for ubiquitination and proteasomal degradation. This difference suggests that host cells and viruses may engage opposing ubiquitin-mediated strategies to modulate PB1 function and innate immune signaling. It is conceivable that KCMF1-mediated degradation of PB1 may occur at an earlier stage of infection, thereby limiting the formation or function of PB1-associated immune evasion complexes. Further studies will be required to elucidate the dynamic interplay between PB1, KCMF1, and MAVS during infection.

In summary, we identify KCMF1 as a novel host restriction factor that suppresses IAV replication by targeting PB1 for ubiquitination-dependent degradation. Disruption of this regulatory axis through mutation of the PB1 K653 residue enhances viral replication and pathogenicity. These findings highlight the importance of site-specific ubiquitination in controlling influenza virus polymerase function and provide new insights into host-virus interactions that may inform future antiviral strategies.

## Data Availability

The data discussed in this publication have been deposited in the Figshare database (https://doi.org/10.6084/m9.figshare.28378925).

## SUPPLEMENTAL MATERIAL

Supplemental material jvi.00481-26-s0001.docx Tables S1 to S3; Fig. S1 to S5.

ASM does not own the copyrights to Supplemental Material that may be linked to, or accessed through, an article. The authors have granted ASM a non-exclusive, world-wide license to publish the Supplemental Material files. Please contact the corresponding author directly for reuse.
