# Supplementary material for: KCMF1-mediated influenza A virus PB1 ubiquitination at K653 regulates viral replication
Source: J Virol. 2026 Jun 15;100(7):e00481-26. doi: 10.1128/jvi.00481-26 (PMC13386863; doi:10.1128/jvi.00481-26)
Supplement: Supplemental material — Tables S1 to S3; Fig. S1 to S5. [file jvi.00481-26-s0001.docx]

Supplementary Materials for

**KCMF1-mediated** **influenza A virus PB1 ubiquitination at K653 regulates viral replication**

Xianfeng Hui ^124#^*, Xiaowei Tian ^5#^, Chang Xue ^23^, Shihuan Ding ^124^, Jiyan Cui ^12^, Aiping Sun ^14^, Wei Lu ^23^, Yunwei Lou ^23^, Shaoju Qian ^14^, Tiesuo Zhao ^14^, Liangwei Duan ^23^ and Hui Wang ^23^*

^1^ Department of Immunology, School of Basic Medical Sciences, Henan Medical University, Xinxiang, China.

^2^ Henan Key Laboratory of Immunology and Targeted Drug, Henan Medical University, Xinxiang, China.

^3^ Henan Collaborative Innovation Center of Molecular Diagnosis and Laboratory Medicine, School of Medical Technology, Henan Medical University, Xinxiang, China.

^4^ Xinxiang Engineering Technology Research Center of Immune Checkpoint Drug for Liver-Intestinal Tumors, Henan Medical University, Xinxiang, China.

^5^ Department of Pathogenic Biology, School of Basic Medical Sciences, Henan Medical University, Xinxiang, China.

* Correspondence: Hui Wang (immuneweb@126.com) (Lead Correspondence) and Xianfeng Hui (Xianfenghui@163.com)

^#^ These authors contributed equally to this work

**This PDF file includes:**

Supplementary Table, Figures and Figure legends

**Supplementary Table**

**Table S1. Candidate E3 ubiquitin ligases for further analysis**

| **Gene ID** | **Gene abbreviation** | **protein name** | **NCBI accession number** |
| --- | --- | --- | --- |
| 79872 | CBLL1 | Cbl proto-oncogene like 1 | NM_001284291.2 |
| 9391 | CIAO1 | cytosolic iron-sulfur assembly component 1 | NM_004804.3 |
| 8451 | CUL4A | cullin 4A | NM_001008895.4 |
| 84259 | DCUN1D5 | defective in cullin neddylation 1 domain containing 5 | NM_001318739.2 |
| 1642 | DDB1 | damage specific DNA binding protein 1 | NM_001923.5 |
| 23741 | EID1 | EP300 interacting inhibitor of differentiation 1 | NM_014335.3 |
| 2521 | FUS | FUS RNA binding protein | NM_001170634.1 |
| 83743 | GRWD1 | glutamate rich WD repeat containing 1 | NM_031485.4 |
| 25831 | HECTD1 | HECT domain E3 ubiquitin protein ligase 1 | XM_017021153.2 |
| 56888 | KCMF1 | potassium channel modulatory factor 1 | NM_020122.5 |
| 54442 | KCTD5 | potassium channel tetramerization domain containing 5 | NM_018992.4 |
| 27339 | PRPF19 | pre-mRNA processing factor 19 | NM_014502.5 |
| 11137 | PWP1 | PWP1 homolog, endonuclein | NM_001317962.2 |
| 5928 | RBBP4 | RB binding protein 4, chromatin remodeling factor | NM_001135255.2 |
| 5931 | RBBP7 | RB binding protein 7, chromatin remodeling factor | NM_001198719.2 |
| 6500 | SKP1 | S-phase kinase associated protein 1 | NM_006930.4 |
| 10273 | STUB1 | STIP1 homology and U-box containing protein 1 | NM_001293197.2 |
| 6923 | TCEB2 | elongin B | NM_007108.4 |
| 9320 | TRIP12 | thyroid hormone receptor interactor 12 | NM_001284214.2 |
| 85363 | TRIM5 | tripartite motif containing 5 | NM_001410958.1 |
| 6737 | TRIM21 | tripartite motif containing 21 | NM_003141.4 |
| 7706 | TRIM25 | tripartite motif containing 25 | NM_005082.5 |
| 10155 | TRIM28 | tripartite motif containing 28 | NM_005762.3 |
| 51366 | UBR5 | ubiquitin protein ligase E3 component n-recognin 5 | NM_001282873.2 |
| 57617 | VPS18 | VPS18 core subunit of CORVET and HOPS complexes | NM_020857.3 |
| 80232 | WDR26 | WD repeat domain 26 | NM_001115113.3 |

**Table S2. The following siRNAs were employed in this study**

| **Name** | **Target sequence** |
| --- | --- |
| siCBLL1-1 | GCACTATAATCAGCCACAT |
| siCBLL1-2 | GGGAATGAGTCCTGGTATA |
| siCBLL1-3 | AGAACGGTATGACTGTAAA |
| siCIAO1-1 | CAAGGACCATTTATGACAT |
| siCIAO1-2 | GCTACCACTTGCATTTGGA |
| siCIAO1-3 | GTGACAGCTGGATCTGCAA |
| siCUL4A-1 | GGAAGAGACTAATTGCTTA |
| siCUL4A-2 | GCATGTGGATTCAAAGTTA |
| siCUL4A-3 | CGAAGGACATCATGGTTCA |
| siCIAO1-1 | CAAGGACCATTTATGACAT |
| siCIAO1-2 | GCTACCACTTGCATTTGGA |
| siCIAO1-3 | GTGACAGCTGGATCTGCAA |
| siCUL4A-1 | GGAAGAGACTAATTGCTTA |
| siCUL4A-2 | GCATGTGGATTCAAAGTTA |
| siCUL4A-3 | CGAAGGACATCATGGTTCA |
| siDCUN1D5-1 | CAATCAAAGTATCGTGTTA |
| siDCUN1D5-2 | CGCTCACAGTTGAATGATA |
| siDCUN1D5-3 | AGAGAAGCCTTGATATTGA |
| siDDB1-1 | GCAGAATCGACTCAATAAA |
| siDDB1-2 | GCCGCATTGAAGTCCAAGA |
| siDDB1-3 | GGCGGCACGTAAAAACCTA |
| siEID1-1 | CAATCGTCTGACCGAAGAA |
| siEID1-2 | GCTGTGATGAGATTATTGA |
| siEID1-3 | GGCGAGGAATTTGATGACT |
| siGRWD1-1 | CTGTCAGGCTTCACCATCT |
| siGRWD1-2 | GACAGAGCTTCCTCTTACA |
| siGRWD1-3 | AGGCCTATGTGCTCTACCA |
| siFUS-1 | CAAGCAGATTGGTATTATT |
| siFUS-2 | CACGGACACTTCAGGCTAT |
| siFUS-3 | GGACAGCAGCAAAGCTATA |
| siKCMF1-1 | GCAGCCTGTTTGTCCAAGA |
| siKCMF1-2 | GCAACAACCAACATAGCTA |
| siKCMF1-3 | GCAGGAGGACAGCTTAATT |
| siKCTD5-1 | GAACGAGACAGCAAAACAT |
| siKCTD5-2 | AGCATCGGCTCCTCTTACA |
| siKCTD5-3 | GTTGGAGGAAGCAGAATTT |
| siHECTD1-1 | CAAGTATATAGCACAGAACA |
| siHECTD1-2 | AAACAAGATTGTAGTCAACT |
| siHECTD1-3 | GATGATGATTATGTGCTAAA |
| siPWP1-1 | ACAGGACGCTTGATGATGA |
| siPWP1-2 | CTACGGGAGTAATGATCAA |
| siPWP1-3 | GCCCAGTGATAATCTTATA |
| siPRPF19-4 | GACAAAAGTTCTGAACAAA |
| siPRPF19-5 | CGATGCCACTATCAGGATT |
| siPRPF19-6 | GACGGACTCATCTTTGGAA |
| siRBBP7-1 | GGAGGAGCGTGTCATCAAT |
| siRBBP7-2 | GCAGAATCCTCACATCATT |
| siRBBP7-3 | GACACCAGGTCCAATACCA |
| siRBBP4-1 | GCAACAAAGACTCCTTCCA |
| siRBBP4-2 | GATACTCGTTCAAACAATA |
| siRBBP4-3 | GCACTTACTTAGTGCTTCA |
| siSKP1A-1 | GCAAGACCTTCAATATCAA |
| siSKP1A-2 | TGAAGATGATGAGAACAAA |
| siSKP1A-3 | GCCAAACAATCTGTGACTA |
| siTRIM5-1 | GAAGTCCATGCTAGACAAA |
| siTRIM5-2 | GGAATCCTGGTTAATGTAA |
| siTRIM5-3 | GCAGAAAGTTGATCATTGT |
| siSTUB1-1 | CTGTGAAGGCGCACTTCTT |
| siSTUB1-2 | GCTCTTCGAATCGCGAAGA |
| siSTUB1-3 | AGCGCTGGAACAGCATTGA |
| siTRIP12-1 | GGGCCATGTTAGAAATCCA |
| siTRIP12-2 | GAAGGCGTTTCTAGGCAAT |
| siTRIP12-3 | GCACCTAGATTGGATAGAA |
| siTRIM28-1 | GGAGCACATTCTGCGCTTT |
| siTRIM28-2 | GGACCATGACCAAGATCCA |
| siTRIM28-3 | GCAACAGTGCTTCTCCAAA |
| siTRIM21-1 | GCAGCACGCTTGACAATGA |
| siTRIM21-2 | GGACAATTTGGTTGTGGAA |
| siTRIM21-3 | GCTTTCTGCTCAAGAATCT |
| siTRIM25-1 | GGGTCAACAGCAAGTTTGA |
| siTRIM25-2 | GCACCATAGACCTCAAAAA |
| siTRIM25-3 | CAGCAAGCTTCCCACGTTT |
| siVPS18-1 | GACGTAAGGATGACGCAAA |
| siVPS18-2 | TCCTCTACGTGAACCGAAA |
| siVPS18-3 | ACTCACTACTGGCCTATCT |
| siWDR26-1 | CTACCAAATTCCGAAATCA |
| siWDR26-2 | GGATGTCATTAGGCTAATA |
| siWDR26-3 | GCAACAGCCTGAATGTCAA |
| siUBR5-1 | GGAACAGGCTACTATTAAA |
| siUBR5-2 | GATGGAGCCTCATTTGATA |
| siUBR5-3 | GGAACTGGATGGTCAGTAT |

**Table S3. The main primers used in this study**

| **Name** | **Sequence（5'-3'）** |
| --- | --- |
| K531R-F: | CTGTCATCAGAAACAATATGATAAAC |
| K531R-R: | CATATTGTTTCTGATGACAGTAAC |
| K553R-F: | GTTGTTCATCAGAGATTACAGGTAC |
| K553R-R: | CTGTAATCTCTGATGAACAACTGAA |
| K577R-F: | GATCATTTGAAATAAGGAAACTGTG |
| K577R-R: | CACAGTTTCCTTATTTCAAATGATC |
| K578R-F: | GATCATTTGAAATAAAGAGACTGTGG |
| K578R-R: | CTCCCACAGTCTCTTTATTTCAAATGA |
| K586R-F: | GCAAACCCGTTCCAGAGCTGGACTGC |
| K586R-R: | GTCCAGCTCTGGAACGGGTTTGCT |
| K612R-F: | GAAGTCTGCCTAAGATGGGAATTG |
| K612R-R: | CAATTCCCATCTTAGGCAGACTTC |
| K635R-F: | GTCAGCCATAGAGAAATTGAATC |
| K635R-R: | GATTCAATTTCTCTATGGCTGAC |
| K653R-F: | CATGGTCCAGCCAGAAACATGG |
| K653R-R: | ACTCCATGTTTCTGGCTGGACCAT |
| K669R-F: | CCTGGATCCCCAGAAGAAATCG |
| K669R-R: | CGATTTCTTCTGGGGATCCAGG |
| K698R-F: | CAATTTATTTGAAAGATTCTTCC |
| K698R-R: | GGGGAAGAATCTTTCAAATAAATTG |
| K736R-F: | GGAAGGATAAGGAAAGAAGAG |
| K736R-R: | CTCTTCTTTCCTTATCCTTCC |
| K737R-F: | CTGGAAGGATAAAGAGAGAAGAGTTC |
| K737R-R: | GAACTCTTCTCTCTTTATCCTTCCAG |
| K745R-F: | GAGATCATGAGGATCTGTTCC |
| K745R-R: | GGAACAGATCCTCATGATCTC |
| K757R-F: | CAGACGGCAAAGATAGGAATTC |
| K757R-R: | GAATTCCTATCTTTGCCGTCTG |
| qRT-PCR-IAV-NP-F | AACGACCGGAATTTCTGGAGAGG |
| qRT-PCR-IAV-NP-R  Uni12 primer | CCGTACACACAAGCAGGCAAGC   AGCAAAAGCAGG |
| QPCR-CBLL1-F | CGAATCATGGATCACACTGACAA |
| QPCR-CBLL1-R | GGTGCAGGTTTCGCTTTGTT |
| QPCR-CIAO1-F | AGGCAATGAACAAGGGGTGG |
| QPCR-CIAO1-R | TTGAGTGGAAGCCGGACAAA |
| QPCR-CUL4A-F | TCAAGAACTTCCGAGACAGACC |
| QPCR-CUL4A-R | GCTCCTCGAGGTTGTACCTG |
| QPCR-DCUN1D5-F | GGGGCTGGAGACAGCTATTG |
| QPCR-DCUN1D5-R | AACCAAGCCAGGCACTTCTT |
| QPCR-DDB1-F | TCAAAAGGATAGCGCTGCCA |
| QPCR-DDB1-R | TGCATTACCAGAGAGCCGTG |
| QPCR-EID1-F | TGATGACTGGGAGGACGACT |
| QPCR-EID1-R | CCAGGGCTGGTTCTCTTGTT |
| QPCR-FUS-F | GCCAGAGCCAGAACAGCTATG |
| QPCR-FUS-R | GCCCGTAAGACGATTGGGAG |
| QPCR-GRWD1-F | CCCATGGAAGCCGAGTCC |
| QPCR-GRWD1-R | GGGGCGCCAGTCTGC |
| QPCR-KCMF1-F | TCCCGACATGAAGGTGTCAG |
| QPCR-KCMF1-R | TGGGTGGTCAGTTGTATGCC |
| QPCR-KCTD5-F | TACTTCCTCACCACTCGGCA |
| QPCR-KCTD5-R | GGCGCCTGTTTCATCCTTGT |
| QPCR-PRPF19-F | GTTTCACCCTTCCCAGGACC |
| QPCR-PRPF19-R | GCCTGTCACAGCACTCTCAT |
| QPCR-PWP1-F | AGAGCTGCAGTTCAGAAGGTAGAG |
| QPCR-PWP1-R | CACTGCCACCACCTTCTTCTT |
| QPCR-RBBP4-F | CGAGCTCTTGCAGCCTCC |
| QPCR-RBBP4-R | CGTCGTCGAAGGCTTCCTTG |
| QPCR-RBBP7-F | GGTCGTCATCTTAGAACAGTGTTTG |
| QPCR-RBBP7-R | ACTGAACGGTAAGACTGGGC |
| QPCR-SKP1-F | AACACCGAACACCATGCCTT |
| QPCR-SKP1-R | GGAACTGGGTCATCATCTCCT |
| QPCR-STUB1-F | CAGGGCAATCGTCTGTTCGT |
| QPCR-STUB1-R | CTTCTGCGCTCTGGGATCG |
| QPCR-TRIP12-F | AGAGGTGCCAATGTCCAACC |
| QPCR-TRIP12-R | TGCCCTAAATGTGACCTTCCTC |
| QPCR-TRIM5-F | GCCCGTGGATTGGGATCTG |
| QPCR-TRIM5-R | CAGGAGTTCCAGGCAGATGG |
| QPCR-TRIM21-F | TCCCCTGTAAAGCCAAACCC |
| QPCR-TRIM21-R | ATCATTGTCAAGCGTGCTGC |
| QPCR-TRIM25-F | TCCAAGAAACCTCCCCCTGT |
| QPCR-TRIM25-R | GAGTTCGGATGTGAGCTGGT |
| QPCR-TRIM28-F | AGGAGGTTCGCAGCTCAATC |
| QPCR-TRIM28-R | CCCCGCTTATTCAGCTCCTT |
| QPCR-UBR5-F | CCAGCTCAATGACAGGTTACG |
| QPCR-UBR5-R | GCAGCATGATTTGGTCCCAC |
| QPCR-VPS18-F | TTTGTAATCCCCAGGCCCCG |
| QPCR-VPS18-R | GCATTCACATACCCCGAGTGG |
| QPCR-WDR26-F | TCCTCATGCTATTGTGAGGATGAA |
| QPCR-WDR26-R | GGCGTCAATTCACAGCGTAG |

**Supplementary Figures and Figure legends**


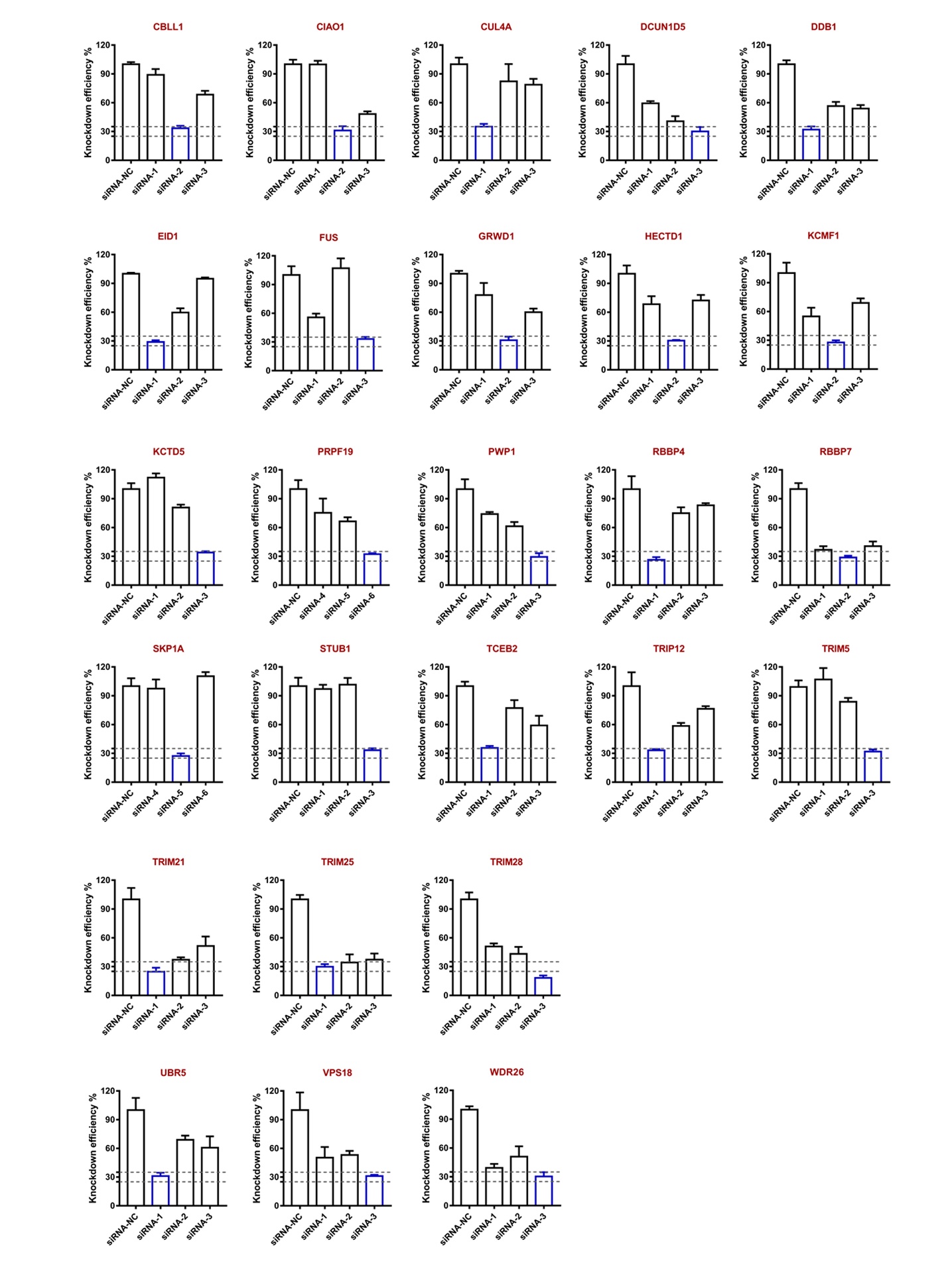


**Supplementary Figure 1 (related to Figure 2). Knockdown efficiency of siRNAs assessed by RT-PCR.** A549 cells were transfected with the indicated siRNAs, and mRNA levels of the target genes were measured by RT-PCR (n = 3).


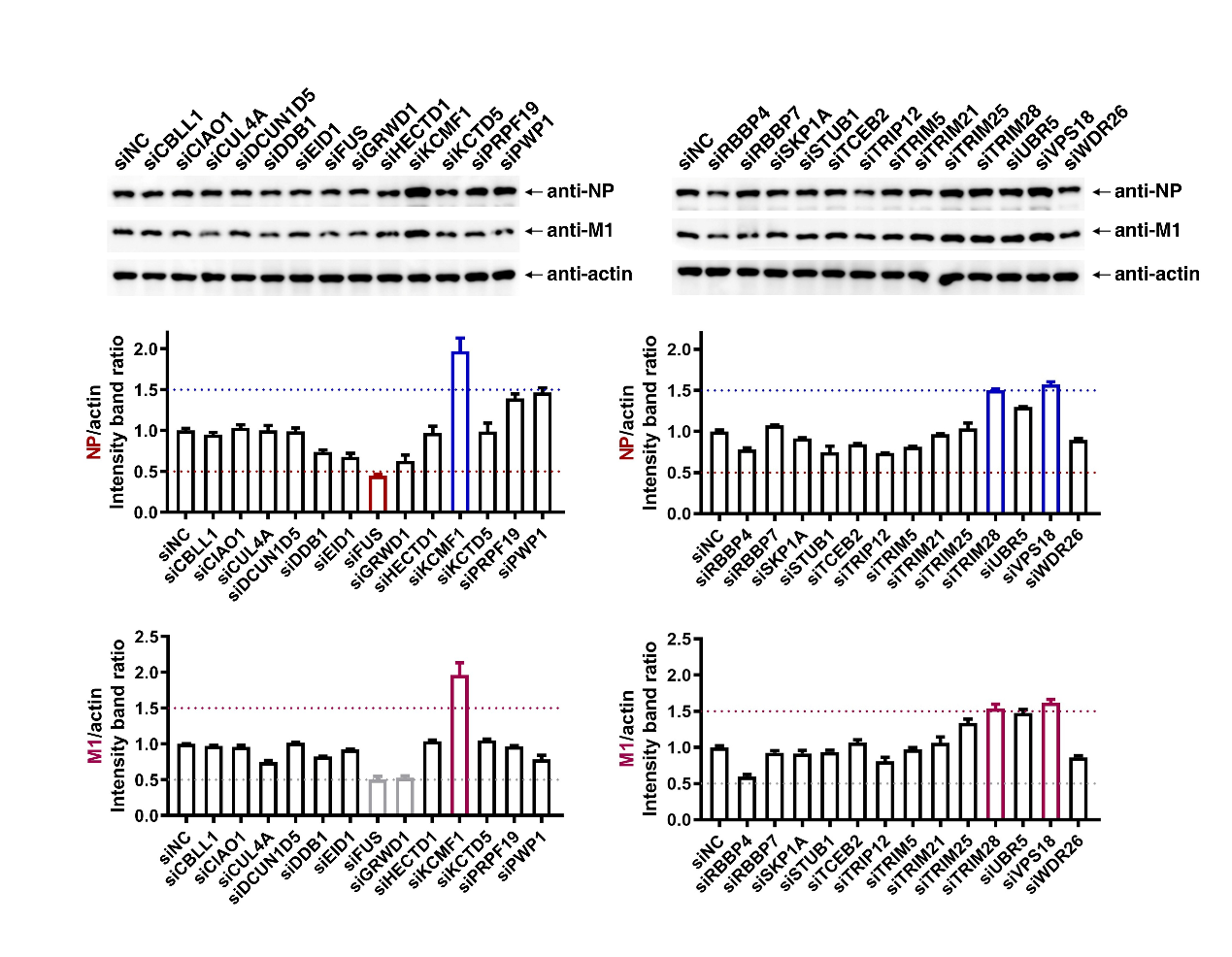


**Supplementary Figure 2 (related to Figure 2). Identification of host factors affecting IAV replication.** A549 cells were transfected with the indicated siRNAs and infected with PR8 virus 24 hpt. Cells were harvested, and levels of viral NP and M1 proteins were analyzed by Western blot. Band intensities were quantified using ImageJ software.


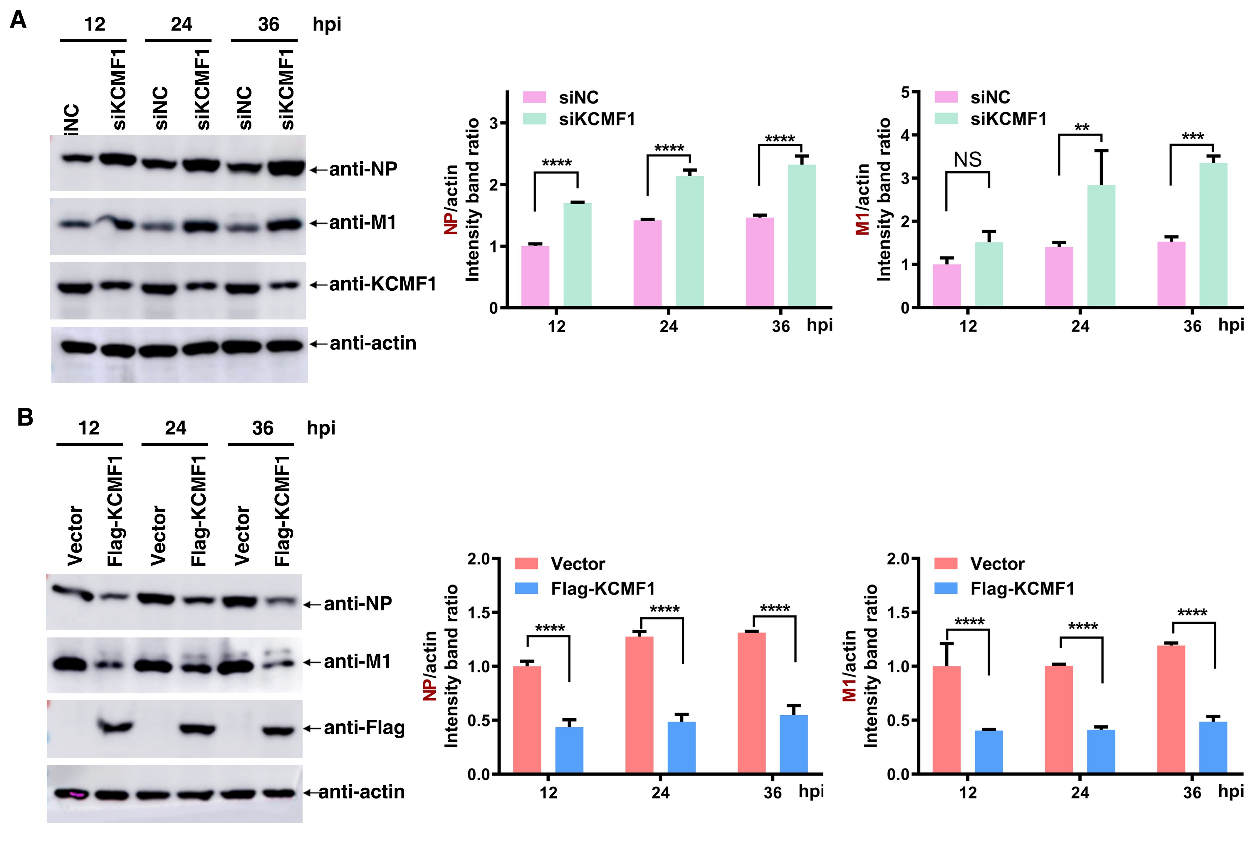


**Supplementary Figure 3 (related to Figure 3). KCMF1 negatively regulates the replication of H1N1 influenza virus.** (A) A549 cells were transfected with the indicated siRNAs and infected with PR8 (H1N1) virus at 24 hpt. Cells were harvested and analyzed by Western blot. (B) A549 cells were transfected with the indicated plasmids and infected with PR8 (H1N1) virus at 24 h post-transfection. Cells were harvested and analyzed by Western blot. Statistical significance was determined using two-way ANOVA. *P < 0.05; **P < 0.01; ***P < 0.001; ****P < 0.0001; NS, not significant.


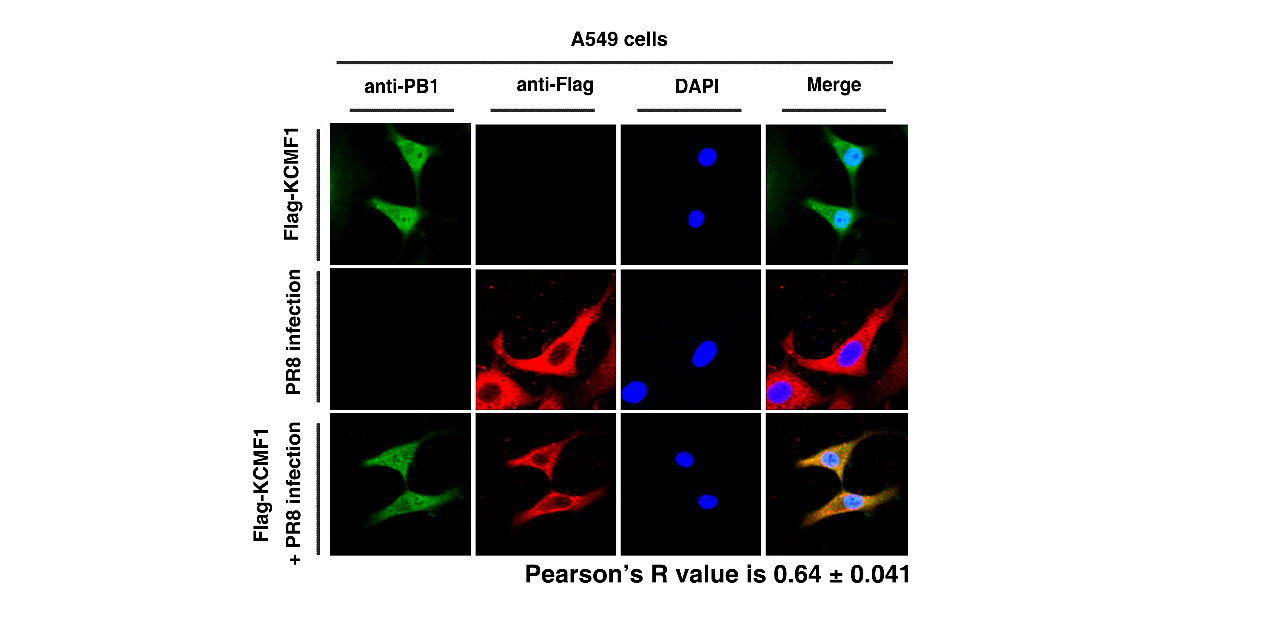
**Supplementary Figure 4 (related to Figure 4). KCMF1 interacts with influenza virus PB1.** A549 cells were transfected with a Flag-tagged KCMF1 expression plasmid and subsequently infected with PR8 (H1N1) virus. Colocalization of Flag-KCMF1 and viral PB1 was analyzed by confocal microscopy. Colocalization was quantified using Pearson’s correlation coefficient (R), calculated with ImageJ software (n = 10).


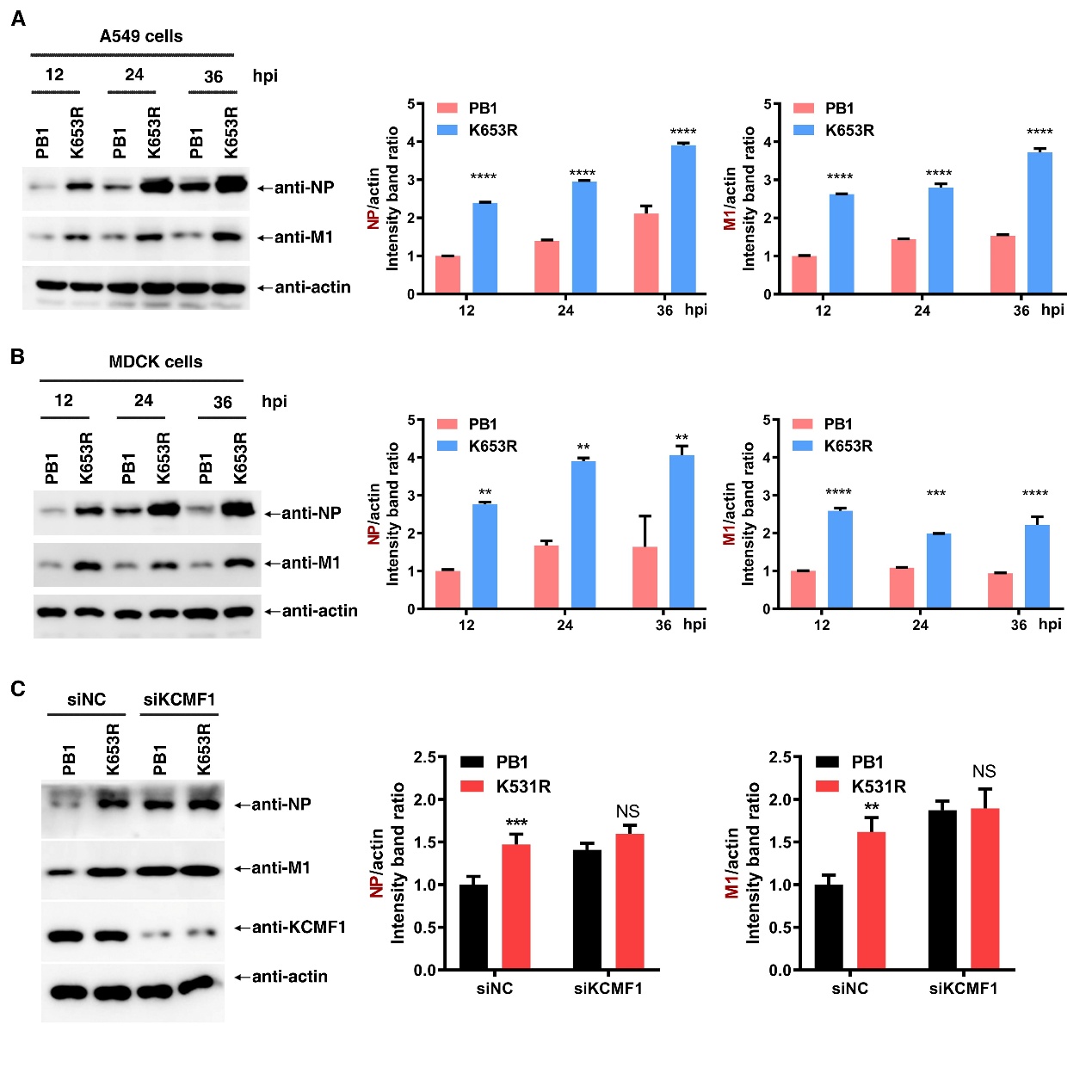


**Supplementary Figure 5 (related to Figure 7). Effects of the PB1 K653 mutation on viral replication in cells.** (A) A549 cells were infected with PR8-WT or PR8-PB1-K653R, and cell lysates were analyzed by Western blot. (B) MDCK cells were infected with PR8-WT or PR8-PB1-K653R, and cell lysates were analyzed by Western blot. (C) A549 cells were transfected with the indicated siRNAs and subsequently infected with PR8-WT or PR8-PB1-K653R at an MOI of 0.05. Cells were harvested at 24 hpi for Western blot analysis. Data are presented as means ± SD from three independent experiments. Statistical significance was determined using two-way ANOVA. *P < 0.05; **P < 0.01; ***P < 0.001; ****P < 0.0001; NS, not significant.
